# Phytochemistry and Pharmacology of the Genus *Equisetum* (Equisetaceae): A Narrative Review of the Species with Therapeutic Potential for Kidney Diseases

**DOI:** 10.1155/2021/6658434

**Published:** 2021-03-05

**Authors:** Thaise Boeing, Karyne Garcia Tafarelo Moreno, Arquimedes Gasparotto Junior, Luisa Mota da Silva, Priscila de Souza

**Affiliations:** ^1^Escola de Ciências Farmacêuticas de Ribeirão Preto, Universidade de Sa˜o Paulo, Ribeira˜o Preto, Sa˜o Paulo, Brazil; ^2^Laboratório de Farmacologia Cardiovascular (LaFaC), Faculdade de Ciências da Saúde, Universidade Federal da Grande Dourados, Dourados, MS, Brazil; ^3^Programa de Pós-Graduação em Ciências Farmacêuticas, Núcleo de Investigações Químico-Farmacêuticas, Universidade do Vale do Itajaí, Itajaí, Brazil

## Abstract

The *Equisetum* genus, Equisetaceae family, is widely distributed worldwide and may be the oldest nonextinct genus on Earth. There are about 30 known species, which are very often used in traditional medicine with diverse applications. This review aimed to compile scientific reports about *Equisetum* species with relevant pharmacological properties and/or therapeutic potential for kidney diseases. Our bibliographic survey demonstrates that the most widespread traditional use of *Equisetum* is as a diuretic, followed by the treatment of genitourinary diseases (kidney diseases, urethritis, kidney stones, and others), inflammation, wound healing, rheumatic diseases, prostatitis, and hypertension. The most popular species from the *Equisetum* genus with medicinal use is *E. arvense* L., whose diuretic effect was confirmed in animal models and clinical trials. The species *E. bogotense* Kunth also demonstrated the beneficial effect of inducing diuresis in both experimental and clinical assays. Several other species have also been studied regarding their therapeutic potential, showing different biological actions. Regarding the chemical composition, it contains many active constituents, such as alkaloids, flavonoids, phenol, phytosterols, saponins, sterols, silicic acid, tannin, triterpenoids, and volatile oils. However, despite the widespread traditional use, many species need to be explored in detail for scientific validation of popular use. Indeed, the species of the *Equisetum* genus have great potential in the management of kidney disorders.

## 1. Introduction

The genus *Equisetum*, belonging to the Equisetaceae family, from Equisetales order and Equisetopsida class, is a genus of perennial plants that reproduce by spores not seeds, widely distributed worldwide, only absent in Australasia and Antarctica [1, 2]. It may be the oldest nonextinct genus on Earth, originating from the end of the Paleozoic era, about 300 million years ago. There are about 30 known species, with the majority consisting of small plants, which rarely reach a meter in height. Its varied species are adapted to grow in temperate, tropical, and cold regions. They are often used in traditional medicine with diverse applications in many countries, having mainly anti-inflammatory and diuretic activities [3].

The species of *Equisetum* genus are known by the common name of “horsetail” in English-speaking countries, “cola de caballo” in Spanish-speaking countries, “prêle des champs” in France, “ackerschachtelhalm” in Germany, “tsukushi” in Japan, and “cavalinha” in Brazil. Its name is of Latin origin, composed of “equi” (horse) and “setum” (tail), that is, horse tail [4–6]. The most popular species from the *Equisetum* genus with medicinal use is *E. arvense* L., which has already demonstrated many biological properties, such as antioxidant, antitumoral, antimicrobial, smooth muscle relaxant, anticonvulsant, sedative, antianxiety, antinociceptive, anti-inflammatory, antidiabetic, diuretic, platelet aggregation inhibitory, osteoblastic response promoting, and antileishmanial effects (for review see [7]). However, many other species are also known for their popular usage and common indications for diuretic purposes and kidney disorders, as will be addressed in this review.

For that, this review's objective was to compile the data found in the literature about *Equisetum* species with relevant pharmacological properties for the treatment of kidney disorders, especially associated with arterial hypertension. We have collected reports from ethno botanical textbooks, and scientific articles from books and journals indexed online in the databases PubMed (https://www.ncbi.nlm.nih.gov/pubmed), Science Direct (http://www.sciencedirect.com/), and Medline (https://www.nlm.nih.gov/bsd/pmresources.html). We will discuss popular uses, toxicological data, phytochemical composition, and pharmacological evidence of these species in the next topics. The main findings are summarized in tables for each theme.

## 2. Traditional Applications and Toxicological Information

Studies that describe this genus's traditional applications, summarized in Table 1, show that the oral administration is the most common route for its use, using the infusion or decoction of the aerial parts as the main method of preparation [1, 9, 42]. The most widespread traditional use of *Equisetum* is as a diuretic [1, 12, 33, 34, 41, 42], followed by the treatment of genitourinary diseases (kidney diseases, urethritis, kidney stones, and others) [10, 16, 25, 26, 33, 34, 37–39], inflammation [10, 20–22, 27, 40], wound healing [21, 28, 39, 41], rheumatic diseases [9, 19, 31, 34, 39], prostatitis [33, 36, 40], and hypertension [1, 21, 27].

Studies compiled in this work pointed out that the most popular species from the *Equisetum* genus with medicinal use is *E. arvense* L., commonly known as “horsetail”, with uses reported in countries like Brazil, Romania, Germany, Serbia, China, Greece, Portugal, Iran, and Thailand. This species is used mainly as diuretic, to treat inflammation, genitourinary diseases, ulcers, wound healing, dermatitis, hemorrhage, hepatitis, prostatitis, musculoskeletal diseases, and others (Table 1) [12, 20–22, 25, 33, 38, 39, 41, 42]. Nevertheless, Baracho et al. [43] evaluated the acute hepatotoxicity of *E. arvense* in rats. The extract at 30, 50, and 100 mg/kg did not show mortality in any of the doses at the end of the 14 days of observation, nor did it alter the serum activities of hepatic enzymes compared to the control group. Instead, it produced benign changes in the hepatic morphology. Moreover, Tago et al. [44] evaluated the toxicity of *E. arvense* in the diet at doses of 0, 0.3, 1, and 3% for 13 weeks in male and female rats. According to the authors, the dosage selections were based on estimated intake for humans, approximately 5 mg/kg daily. No toxicity was detected related to clinical signs, body weight, urinalysis, hematology, serum biochemistry data, organ weights, and histopathological findings. Still, remedies containing *E. arvense* are not recommended during pregnancy or breastfeeding since little information is available on their safety [45]. Indeed, the species contains thiaminase, an enzyme that destroys thiamine (vitamin B1), and, with long-term use, could lead to vitamin deficiency, a possible cause of neurotoxicity [46].

Another well-cited species is *E. telmateia* Ehrh., known as “great horsetail” [47], used to treat rheumatism, broken bones, genitourinary diseases, prostatitis, gastrointestinal disorders, inflammation, and hypertension, in addition to its use as diuretic and expectorant in countries like Turkey, Portugal, Iran, and Spain [1, 34, 36, 37, 40]. However, little information is available about its safety. *E. giganteum* L., commonly called “giant horsetail” [10], is another widespread species mainly in Latin American countries like Brazil, Bolivia, Chile, and Peru, traditionally used to treat diarrhea, heartburn, genitourinary disorders, inflammation, rheumatic diseases, obesity, and as a diuretic [8–11, 18, 28]. Despite its widespread traditional use, no *in vivo* toxicity studies were conducted so far; however, Alavarce et al. [10] showed no cytotoxic potential of the hydroalcoholic extract of *E. giganteum* aerial parts (50, 25, 16, 8, and 4 mg/mL) on human palatal epithelial cells and human monocytes using MTT assay.

Similarly to *E. giganteum*, the species *Equisetum bogotense* Kunth also grows in Chile, where it is widely used in traditional medicine as a diuretic [48]. As far as we know, there are no cytotoxic studies carried out on animals with this species; however, *E. bogotense* was subjected to a clinical study where 25 healthy patients received their infusion at an equivalent dose of 0.75 g of the plant per person, daily, for two consecutive days, but no adverse reactions were detected (e.g., diarrhea, asthenia, dizziness, colic, vomiting, palpitations, and hypotension) [18].

*Equisetum myriochaetum* Schlecht. and Cham. is a plant distributed in Mexico, where it is popularly known as “cola de caballo” [49] and traditionally used for the treatment of diabetes type 2 and kidney disease [14–17]. Regarding toxicological information, Téllez et al. [49] have shown that extracts from *E. myriochaetum* aerial parts had no acute toxicity detected in *Drosophila* or in the human micronucleus test *in vitro* performed with cultured lymphocytes, emphasizing that this species is not genotoxic.

Moreover, *Equisetum debile* Roxb. ex Vaucher, also known as “horsetail”, is widely distributed throughout Thailand. The local population has been used as a diuretic, wound muscle relaxant, hair growth stimulant, and anti-hair loss treatment. The *E. debile* extracts showed no cytotoxicity on dermal papilla cell line (1 to 500 *μ*g/mL) and no irritation on chorioallantoic membrane of hen's eggs (0.5%) [41], but further studies are needed to ensure their safety.

*Equisetum palustre* L., known as “marsh horsetail” [50], has been traditionally used in Turkey for peptic ulcer, hemorrhoids, and kidney stones treatment [35]. Despite this, *E. palustre* has been known for its toxicity for livestock, which is related to the presence of thiaminase and the alkaloids palustrine and nicotine [50]. Besides, Milovanović et al. [24] showed that *E. palustre*, as well as *E. arvense* L., *E. sylvaticum* L., *E. fluviatile* L., and *E. telmateia* Ehrh. extracts (62.5 *μ*g/ml), showed some genotoxicity, presenting a higher incidence of micronucleus formation than that of the control.

## 3. Phytochemistry Data of *Equisetum* Genus

In this topic, we reviewed the phytochemistry data available on the plants' species of *Equisetum* genus (Table 2), which have already been presented for their traditional applications. Chemical structures of the compounds identified in the *Equisetum* genus followed by their names, molecular weights, and references are shown in Figure S1 (Supplementary Material).

*E. arvense* L. contains various chemical compounds such as silicic acid, linoleic acid, oleic acid, stearic acid, linolenic acid and traces of alkaloids (e.g., equisetin, nicotine, palustrine, and palustrinine), glucoside, flavonoids, saponosides, triterpenoids, phytosterols, calcium carbonate, potassium sulfate, potassium chloride, manganese chloride, iron, manganese, and calcium phosphate [39, 52]. Indeed, Veit et al. [51] isolated two styrylpyrone glucosides (3′-deoxyequisetumpyrone and 4′-*O*-methylequisetumpyrone) from the MeOH extract from the rhizomes of *E. arvense.* Besides, in the hepatoprotective activity-guided fractionation of the MeOH extract from the aerial parts of *E. arvense* L. performed by Oh et al. [25], the bioactive EtOAc fraction was subjected to octadecyl-functionalized silica gel flash column chromatography resulting in the isolation of two phenolic petrosins (onitin and onitin-9-*O*-glucoside) and four flavonoids (apigenin, luteolin, kaempferol-3-*O*-glucoside, and quercetin-3-*O*-glucoside). Similarly, Mimica-Duki [52] evaluated the phenolic composition of three different extracts (EtOAc, n-BuOH, and H_2_O). In this study, quercetin 3-*O*-glucoside (isoquercitrin) was the main compound in the EtOAc identified by high-performance liquid chromatography with diode-array detection (HPLC-DAD). At the same time, apigenin 5-*O*-glucoside and kaempferol 3-*O*-glycoside were detected in considerable amounts. The n-BuOH extract showed higher amounts of isoquercitrin and di-*E*-caffeoyl-meso-tartaric acid, while the aqueous extract had di-*E*-caffeoyl-meso-tartaric acid and also two phenolic acids detected. Also, Ganeva et al. [53] isolated terpenoids (taraxerol, *β*-amyrin, germanicol, *α*-amyrin, ursolic acid, oleanolic acid, betulinic acid, taraxasterone, and *ψ*-taraxasterone) and some sterols (isobauerenol, epicholestanol, cholesterol, sitosterol, and 28-isofucosterol) from the aerial parts of this species using preparative thin layer chromatography (PTLC) and gas chromatography-mass spectrometry (GC-MS). In this sense, Fons et al. [55] investigated the volatile profile of fresh aerial parts of *E. arvense*, also using GC-MS. The plant contained a great biodiversity of isoprenoid flavor precursors (3-hydroxy-7,8-epoxy-*β*-ionol, (*E,E*)-pseudoionone, and 3-oxo-*α*-ionol), as well as odorous benzenic derivatives (phenylethanal, 2-phenylethanol, benzaldehyde, and homovanillic acid).

The hydroalcoholic extract of *E. arvense* sterile stems was also characterized by Milovanović et al. [24]. The authors have found the nonmalonylated quercetin 3-*O*-glucoside and the free aglycone quercetin as the major constituents, but quercetin 3-*O*-(6″-*O*-malonylglucoside), 5-*O*-caffeoyl shikimic acid, monocaffeoyl-meso-tartaric acid, and dicaffeoyl-meso-tartaric acid were also detected. Besides, Gründemann et al. [22] have also focused on the *E. arvense* extract's phytochemical analysis on identifying flavonoids and other polar phenolics. A decoction produced by boiling a part of dry plant material with nine parts of ethanol for 4 h was subjected to the HPLC method to separate the phenolic compounds in the extract later identified by liquid chromatography-mass spectrometry (LC-MS). The major constituents were mono-, di-, and triglycosides of kaempferol, quercetin, apigenin, genkwanin, and protogenkwanin well as mono- and dicaffeoyl-tartaric acid. Besides, three phenolic glycosides, equisetumoside A, equisetumoside B, and equisetumoside C, were isolated from the water-soluble extract of fertile sprouts of *E. arvense* L., together with uridine, inosine, 2′-deoxyinosine, 2′-deoxycytidine, tryptophan, thymidine, 5-carboxy-2′-deoxyuridine, coniferin, and kaempferol 3-*O*-*β*-D-sophoroside-7-*O*-*β*-D-glucopyranoside, by Chang et al. [54].

As described in the clinical study conducted by Lemus et al. [18], only a few chemical screenings have been published in the late 1980s and early 1990s demonstrating the presence of *β*-sitosterol, silicic anhydride, flavonoids, and coumarins, as well as isokaemferide and kaempferol derivatives in *E. bogotense* HBK. Besides, more recently, Tipke et al. [56] identified and quantified the alkaloids present in this species by hydrophilic interaction liquid chromatography high‐performance liquid chromatography tandem mass spectrometry (HILIC HPLC‐MS/MS) in electrospray ionization (ESI). The plant material was powdered using a standard electric coffee grinder and submitted to alkaloids extraction using sulphuric acid. The presence of nicotine, palustrine, and palustridiene was detected; however, only two out of five samples were positive for the compounds. Therefore, the authors stated that more data is necessary to have a clearer picture of the occurrence of alkaloids in this species.

Xu et al. [58] reported the isolation and characterization of three new megastigmane glucosides and four known constituents from the whole plant of *E. debile*. The dried whole plant of *E. debile* was extracted with CHCl_3_ and 70% EtOH. After solvent evaporation, the EtOH extract was partitioned with AcOEt and BuOH, subjected to column chromatography (CC). The AcOEt fraction afforded blumenol A and corchoinoside C, while BuOH-soluble extract afforded sammangaoside A, debilosides A–C, and (3S,5R,6R,7E,9S)‐9‐[(*β*‐D‐glucopyranosyl)oxy]megastigm‐7‐ene‐3,5,6‐triol. Moreover, Tan et al. [57] investigated this plant's chemical constituents obtaining 12 compounds. The whole dried plant of *E. debile* was powdered, extracted with EtOH, and posteriorly partitioned. The EtOAc fraction yielded the new phenylhexane debilitriol, the new alkaloid equisetumine, guaiacylglycerol-*β*-coniferyl ether, and the compounds 5-hydroxymethyl-2-furfuraldehyde, coumaric acid, *p*-hydroxybenzoic acid, and ferulic acid. In contrast, the compounds (+)-lariciresinol 9-*O*-*β*-D-glucopyranoside, 8-*O*-4′ neolignan glucoside debilignanoside, equisetumoside B, (+)-isolariciresinol-3a-*O*-*β*-D-glucopyranoside, and thymidine were obtained from BuOH fraction. Besides, Kanchanapoom et al. [59] described the isolation of chemical constituents from the MeOH extract from aerial parts of *E. debile,* including megastigmane glucosides (macarangioside *D*, sammangaoside A, (3S, 5 R, 6S, 7 E, 9S)-megastigman-7-ene-5,6-epoxy-3,9-diol 3,9-*O*- *β*-D-diglucopyranoside, (6R,9S)-3-oxo-a-ionol 9-*O*-*β*-D-glucopyranoside, and debiloside B), flavonoid glycosides (kaempferol 3-*O*-sophoroside, kaempferol 3,7-*O*- *β*-D-diglucopyranoside, kaempferol 3-*O*-sophoroside-7-*O*- *β*-D-glucopyranoside), a phenylethanoid glucoside (phenylethyl *O*- *β* -D-glucopyranoside), an aliphatic glucoside (*Z*)-3-hexenyl *O*- *β*-D-glucopyranoside), a neolignan glucoside ((7S, 8 R)-dehydrodiconiferyl 4-*O*- *β*-D-glucopyranoside), and amino acid (L-tryptophan).

Francescato et al. [60] have evaluated the phenolic composition of the hydroalcoholic extract of *E. giganteum* stems by LC-DAD and LC–ESI-MS/MS allowing the characterization of 12 compounds (Table 2). Caffeic acid derivatives, flavonoids, and styrylpyrones were detected; in addition, the phenolic components quercetin-3-*O*-(caffeoyl)-glucoside and 3-hydroxyhispidin-3,4′-di-*O*-glucoside were reported for the first time in the *Equisetum* genus, while the most abundant flavonoids were kaempferol derivatives. Similarly, Alavarce et al. [10] identified 13 constituents in the hydroalcoholic extract from the aerial parts of *E. giganteum* by reversed-phase ultra-high-performance liquid chromatography (RP-UHPLC), ultraviolet–visible spectroscopy (UV-Vis), and MS/MS^n^ spectra analysis, also detecting styrylpyrones and flavonoid glucosides derivatives of quercetin and kaempferol. Moreover, the alkaloid palustridiene was the only one found in this species in the plant's acid extract in the study conducted by Tipke et al. [56].

The hydroalcoholic extract from aerial parts of *E. hyemale* L. was characterized by Queiroz et al. [61] by spectrophotometric and HPLC methods with pulsed amperometric detector (PAD) analyses. Phenolic compounds were detected and identified as gallic acid, tannic acid, chlorogenic acid, and caffeic acid. Moreover, Jin et al. [62] identified 9 compounds in the n-BuOH fraction obtained from EtOH extract of *E. hyemale* aerial parts by CC. The authors obtained 8 known compounds, with kaempferol-3-sophoroside-7-*O*-*β*-D-glucopyranoside and kaempferol-7-*O*-*α*-L-rhamnoside-4′-*O-* *β*-D-glycopyranoside being first reported to be isolated from this plant, and a new phenyl glycoside, (2-(sophorosyl)-1-(4-hydroxyphenyl) ethanone).

Regarding *E. myriochaetum*, the compounds kaempferol-3-*O*-sophoroside, kaempferol-3,7-di*O*-*β*-glucoside, kaempferol-3-*O*-sophoroside-4′-*O*- *β*-glucoside, and caffeoyl-methylate-4-*β*-glucopyranoside were isolated by Wiedenfeld et al. [63] and Cetto et al. [66] from the water-soluble portion of the aerial parts of this species. Besides, Camacho et al. [64] also isolated the constituents pinocembrin, chrysin, *β* -sitosterol, *β* -D-glucosylsitosterol, *β* -D-glucose, and fatty acids.

As mentioned before, Tipke et al. [56] analyzed the alkaloids present in *Equisetum* species using HILIC‐HPLC‐ESIpos‐MS/MS approach. Interestingly, the highest levels of alkaloids were detected in *E. palustre* samples (close to 1 g alkaloid per kg), which were mainly dominated by palustrine and palustridiene. Similarly, Cramer et al. [50] screened twenty-two *E. palustre* samples by the same method. Although the alkaloids' content and distribution suffered variability, palustrine and palustridiene were the main compounds found as well. In addition, another four palustrine-like and three palustridiene-like alkaloids were detected, but only four of them were identified, being N^5^-formylpalustrine, N^5^-Acetylpalustrine, 18-deoxypalustrine, and N^5^-formylpalustridiene. Moreover, two related compounds were detected (myricoidine and spermidine). Nevertheless, in the bioassay-guided fractionation study using ethanol (EtOH)-induced ulcer model in rats, Gurbuz et al. [35] have obtained, by high-speed centrifugal countercurrent chromatography (HS-CCCC) technique, three fractions from the n-BuOH extract of the aerial parts of *E. palustre*, identifying for the first time in this species the compound kaempferol 3-*O*-1″-*β*-D-glucopyranosyl-3-*O*-1‴-*β*-D-glucopyranoside. Posteriorly, Wei et al. [65] have also isolated this compound, together with 4-*O*-(*p*-coumaroyl)shikimic acid, luteolin-7-*O*-*β*-D-glucopyranoside, quercetin-3-*O*-*β*-D-glucopyranoside, apigenin-5-*O*-*β*-D-glucopyranoside, genkwanin-5-*O*-*β*-D-glucopyranoside, luteolin, apigenin, and genkwanin using MSPD extraction and UHPLC–MS/MS. It was also observed that the content of flavonoid was markedly higher than that of flavonoid aglycones. Milovanović et al. [24] studied the hydroalcoholic extract from the sterile stems of this species as well. The predominant constituents were kaempferol glycosides, such as kaempferol 3-*O*-rutinoside-7-*O*-sophoroside, kaempferol 3-*O*-rutinoside-7-*O*-glucoside, and kaempferol 3-*O*-glucoside, but other compounds were detected in smaller amounts.

Interestingly, in the same abovementioned study, the HPLC profile of *E. telmateia* hydroalcoholic extract of the sterile stems showed predominance of kaempferol 3,7-*O*-diglucoside and 5-*O*-caffeoyl shikimic acid; however, a relatively high content of acetylated flavonoid derivatives such as kaempferol-3-*O*-(6″-*O*-acetylglucoside), kaempferol 3-*O*-(6″-*O*-acetylglucoside)-7-*O*-glucoside, and kaempferol 3-*O*-(6″-*O*-acetylglucoside)-7-*O*-rhamnoside has been found, so far exclusively, in this species of the genus. Regarding the aerial parts of *E. telmateia*, analysis of the aqueous extract and the ethyl acetate fraction by HPLCPAD-ESI/MS performed by Correia et al. [36] allowed the identification of major phenolic compounds such as flavan-3-ol, kaempferol, and phenolic acid derivatives. Retention characteristics and UV spectra also allowed the identification of protocatechuic and *p*-hydroxybenzoic acids and the presence of various caffeic acid derivatives. Further identities assigned to the ethyl acetate fraction compounds from the infusion of *E. telmateia* are displayed in Table 2.

Finally, in the previously mentioned study carried out by Fons et al. [55] on the volatile profile of fresh aerial parts of *Equisetum* species, *E. palustre* showed forty-four volatile organic compounds, mostly lipidic derivatives (1-octen-3-ol, (*E*)-2-hexenoic acid) and odorous compounds ((*E*)-2-hexenal, (*Z*)-3-hexenol, hexanol, and (*E*)-2-nonenal), while linalool was the only terpenic derivative identified. *E. telmateia* was dominated by a large number of isoprenoid derivatives (3-hydroxy-7,8-epoxy-*β*-ionol and 3-hydroxy-*α*-ionone) as well as one benzenic derivative (3-methoxy-4-hydroxystyrene, also called 4-vinylguaiacol). The major volatile constituents in *E. hyemale* derived from the lipidic pathway ((*E*)-2-heptenal) and shikimic pathway ((*E*)- and (*Z*)-ferulic acid isomers, 4- vinylguaiacol, and isovanillin), while *E. ramosissimum* showed the highest amount of shikimic derivatives ((*E*)-ferulic acid, 4-vinylguaiacol, (*Z*)-ferulic acid, and 2-phenylethanol), but other studies on the chemical composition of the last species mentioned have not been found.

## 4. Pharmacological Potential of *Equisetum* Genus

### 4.1. Species with Therapeutic Potential for Kidney Diseases

Carneiro et al. [13] described the diuretic effect of a standardized dried extract of *E. arvense* (EADE) by monitoring 36 healthy male volunteers' water balance. Through four repeated days, the authors administered EADE (900 mg/day), placebo (corn starch, 900 mg/day), or hydrochlorothiazide (25 mg/day), separated by a 10-day washout period. The *E. arvense* extract was able to significantly induce diuresis similar to the hydrochlorothiazide group, without triggering important variations in the elimination of electrolytes. The clinical checkups and laboratory assessments showed no changes before or after the test, suggesting that the extract of *E. arvense* is safe for acute use. However, despite the promising results described herein, further research is required to elucidate its diuretic action mechanism.

The ability of the ethanol extract from the roots of *E. arvense* to impact the urinary bladder activity in rats was studied by Zhang et al. [67], by treating them with a standard diet containing 0.2% of the extract. After 3 weeks, cystometry with 0.2% acetic acid solution was done and bladder activity was recorded; in addition, blood pressure, body weight, and adenosine triphosphate (ATP) were measured before and after the stimulation. The control group results showed that during cystometry with acetic acid, the time interval between urinary bladder contractions was shorter, and maximum bladder contraction pressure was much greater. In contrast, the changes observed were lower in the *E. arvense* group. Furthermore, the plasma adrenaline and noradrenaline levels were reduced in the *E. arvense* group compared to the control group. Besides, the increase in the levels of urinary ATP was smaller in rats treated with *E. arvense* extract compared to the control group. Therefore, it was demonstrated that the ethanolic extract of root from *E. arvense* influences urinary bladder activity and can treat urinary bladder disorders.

Gažová et al. [68] performed a clinical study designed to evaluate the efficacy and security of CELcomplex^®^, a preparation comprising a mix of *Cucurbita pepo* L. seed extract, *Equisetum arvense* L., and *Linum usitatissimum* L., on stress urinary incontinence in female patients recruited from 20 urological and gynecological patient clinics in Slovakia. Interestingly, after 12 weeks of treatment (625 mg, two pills for the first 14 days and one pill daily until the end of the treatment), the patients presented a 30% improvement in urinary incontinence episodes, 40% improvement in day-time urination frequency, and 64% gain in nocturnal urinary frequency. However, some side effects were reported, including headache, flatulence, and gastrointestinal discomfort. Indeed, further studies may be needed to determine each plant's isolated effect in this combination and the effectiveness and efficacy of this phytotherapy in other populations.

Recently, the diuretic efficacy of another herbal mixture containing *E. arvense* was also evaluated clinically. Perna et al. [69] assessed the effectiveness of 4 and 8 weeks of supplementation with highly standardized formula, with *Fraxinus ornu*s L. plus *Ananas comosus* L*., Betula pendula* R., *Equisetum arvense* L., *Urtica dioica* L., and *Pilosella officinarum* L. Vaill. dry extract, on the state of hydration and bloating sensation in subjects with high and moderate extracellular water. In this study, 19 women with extracellular water over 45% completed the study and their data were analyzed at baseline, at 30 and 60 days. Bioimpedance, short form with 36 questions, and anthropometric parameters were assessed. The extracellular water decreased at 30 and 60 days, as well as the fat mass. Improvement of free fat mass was measured but not on the bloating sensation survey at 60 days. Again, similarly to Gažová et al. [68], further studies may be needed to determine each plant's isolated effect and the efficacy of this herbal mix in other populations.

Another study evaluating the botanical formulation containing *Herniaria glabra* L., *Agropyron repen*s, *Equisetum arvense* L., and *Sambucus nigra* L. was undertaken to explore the preventive role of this combination in an experimental model of nephrolithiasis induced by the oral treatment with 0.75% ethylene glycol (EG) and 1% ammonium chloride for three days, followed by 15 days with only EG. The groups received different doses of the formulation, ranging from 30 mg/kg to 500 mg/kg. The results revealed that the group treated with 125 mg/kg of the formulation had a significant lower calcium oxalate crystals deposits amount when compared with the placebo-only treated group. All the doses significantly diminished the quantity of microcalcifications and reduced the number of kidneys affected by subcapsular fibrosis. Besides, the doses of 125 mg/kg and 500 mg/kg were able to induce diuresis. Although the study has not explored the effects of the species in isolation, this formulation's potential is evident and significant [70]. Indeed, more studies are needed to investigate the mechanisms responsible for the effect and the safe use of these preparations.

Although the main purpose of this review is to discuss the scientific findings of the different *Equisetum* species, it is important to mention that some compounds isolated from these species have already revealed beneficial actions regarding their ability to induce diuresis. In this context, the flavonoid luteolin, found in the aerial parts of *E. arvense* and *E. palustre*, induced both diuretic and natriuretic effect in normotensive and hypertensive rats, without interfering with urinary pH, K^+^, or Cl^−^ levels. This study also showed the involvement of muscarinic acetylcholine receptors for luteolin renal effects [71]. Besides, the diuretic activity of isoquercitrin (quercetin 3-*O*-glucoside), a compound also found in *E. arvense* aerial parts, was studied by Gasparotto et al. [72]. The flavonoid showed diuretic activity in a 7-day repeated-dose study in spontaneously hypertensive rats (SHR). Isoquercitrin (10 mg/kg, p.o.) increased Na^+^ excretion and presented K^+^-sparing effects similar to spironolactone. Those effects were related to angiotensin-converting enzyme inhibition; increased bioavailability of bradykinin, PGI2, nitric oxide; and inhibitory effect on renal Na^+^/K^+^-ATPase activity. Moreover, the treatment with oleanolic acid (3*β*-hydroxy-olea-12-en-28-oic acid) at 60 mg/kg/day for 4 weeks has demonstrated antihypertensive effect in L-NAME-induced hypertension in rats by promoting diuresis and increasing Na^+^ excretion compared to the L-NAME group, in addition to inducing nephroprotection [73].

Extracts from *E. giganteum* and *E. bogotense* have demonstrated a diuretic activity in laboratory animals [18, 48]. Further, the diuretic activity of *E. bogotense* was also evaluated in healthy volunteers through their water balance by Lemus et al. [18]. In addition to biochemical parameters, such as urinary electrolyte concentration, urine density, osmolarity, and pH value, clinical observations on arterial pressure and any adverse reactions were also determined. A 10% *E. bogotense* infusion showed a significant diuretic effect in healthy subjects compared to the control group. Besides, none of the volunteers reported any evidence of adverse reaction, nor did they show any change in blood pressure. Urinary electrolytes showed a significant increase in Na^+^, K^+^, and Cl^−^ excretion compared to the control group, but inside the usual physiological values. Despite the great variation in diuresis excretion levels of the subjects, the study results suggest that an infusion of *E. bogotense* could be used to induce diuresis. As mentioned before, little information is available regarding the chemical composition of this species; only the alkaloids nicotine, palustrine, and palustridiene were identified. Of those, nicotine was described as antidiuretic [74], while the other alkaloids' effect remains unknown.

Gutiérrez et al. [75] proved that the chloroform extracts of some species of *Equisetum* genus, including the *E. fluviatile*, *E. hyemale* var. *affine*, *E. giganteum*, and *E. myriochaetum*, at dose of 50 mg/kg, presented acute diuretic activity in CD1 strain mice using hydrochlorothiazide, spironolactone, and furosemide as standard drugs for comparison (given at a dose of 25 mg/kg). It was found that the most active plant was *E. hyemale* var. *affine*, followed by *E. fluviatile*, *E. giganteum*, and *E. myriochaetum*, producing an effect similar to hydrochlorothiazide in relation to the excretion of Na^+^, K^+^, and Cl^−^. Besides, gallic acid, found in the aerial parts of *E. hyemale*, also exhibited a diuretic effect when orally given to normotensive and hypertensive rats, an effect associated with increased Na^+^ and Cl^−^ levels in the urine. This study also showed the endogenous prostanoid generation's involvement in the renal effects induced by gallic acid [76]. Moreover, caffeic acid, found in the species *E. giganteum*, *E. hyemale*, and *E. telmateia*, induced diuresis in normotensive rats, associated with an increased urinary Na^+^ and K^+^ elimination and a urinary Ca^2+^-sparing effect. In agreement with these findings, this study also demonstrated, by using an *in vitro* assay of urinary calculus formation, that the caffeic acid reduced the number of the monohydrate and dihydrate forms of calcium oxalate crystals formed in the urine, suggesting the diuretic plus antiurolithiatic effect of this compound [77]. On the other hand, it is important to mention that kaempferol, a compound found in many species of the *Equisetum* genus, was studied by Cechinel-Zanchet et al. [78], but did not present diuretic action.

A last compound of interest found in the species *E. giganteum*, *E. ramosissimum*, and *E. debile* is ferulic acid, which has been reported as nephroprotective in a great number of studies conducted on animals. This phenolic acid has been shown to reduce liver and renal oxidative damage induced by cadmium [79], as well as hyperglycemia- [80], lipopolysaccharide- [81], or methotrexate- [82] induced kidney nephrotoxicity by attenuating inflammation process and reducing oxidative stress. For instance, Alam et al. [83] have shown that chronic treatment with ferulic acid (50 mg/kg) also reduced systolic blood pressure of SHR animals and Wistar rats receiving L-NAME for 8 weeks. This compound was able to reduce left ventricular diastolic stiffness and attenuate inflammatory cell infiltration, ferric iron accumulation, and collagen deposition in left ventricles and kidneys in both models of hypertension. Moreover, ferulic acid improved both endothelium-dependent relaxation in isolated thoracic aortic rings and antioxidative balance in the heart and kidneys. Finally, we can suggest that these compounds, at least in part, seem to contribute to the pharmacological effects described by these species. However, many compounds have not yet been the subject of studies that specifically target these biological activities, so there is a fruitful field to be explored in the future.

## 5. Other Properties of Pharmacological Interest

Do Monte et al. [84] described the antinociceptive and anti-inflammatory effects of hydroalcoholic extract of stems from *E. arvense* in mice. Briefly, the results of the present study demonstrated that the extract exhibits an antinociceptive effect in chemical models of nociception. In agreement with these results, Steinbor et al. [85] verified the anti-inflammatory properties of *E. arvense* through its silica content, revealing that silica-rich horsetail preparations suppress lymphocytes' activation and proliferation by an interleukin-2-dependent mechanism and a downregulation of interferon gamma (IFN-*γ*). Interestingly, analytical profiling by HPLC-UV-MS and bioactivity testing revealed significative immunosuppressive concentrations of a component that was identified as isoquercitrin. Thus, the authors concluded that both silica and isoquercitrin are active compounds of horsetail preparations. The anti-inflammatory and antinociceptive effects of *E. arvense* have also been investigated in clinical studies. In one of them, five herbs (including *E. arvense*) plus thiamine reduced the pain and improved functional mobility in patients with pain [86]. The complex of 5 herbs, plus vitamin B1, was well tolerated. The results suggest that the blend should be considered a valuable alternative treatment in managing chronic musculoskeletal pain. However, the exact contribution of *E. arvense* in these clinical results still needs to be quantified.

*E. arvense* has also been described as a promising source of anticancer compounds and its ethanol extract showed a cytotoxic effect on HeLa (cervical cancer cells), HT-29 (colorectal adenocarcinoma), and MCF7 (breast cancer) cell line [87]. Moreover, Alexandru et al. [88] reported that the aqueous extract from sterile stems of *E. arvense* exerted concentration dependent cytotoxic effects on human leukemic cells (U937 cells). The results from Al Mohammed et al. [89] demonstrated that the ethanolic extract of *E. arvense* presented cytotoxicity and decreased the cell viability of adenocarcinomic human alveolar basal epithelial cells (A549 cells). Recently, Bhat et al. [90] studied the cytotoxic and suppressive action of the ethanolic extract of *E. arvense* against human pancreatic carcinoma cell line ASPC-1. The extract showed potential cytotoxicity and reduced the cellular proliferation of these cells. Similarly, a very strong antimicrobial action of volatile constituents of the *E. arvense* was reported by Radulović et al. [91] against the bacteria *Staphylococcus aureus*, *Escherichia coli*, *Klebsiella pneumoniae*, *Pseudomonas aeruginosa*, and *Salmonella enteritidis* and the fungi *Aspergillus niger* and *Candida albicans*. Remarkably, the essential oil of *E. arvense* is shown to possess a broad spectrum of a robust antimicrobial activity against all tested strains.

*E. arvense* for the treatment of osteoporosis has been extensively studied, mainly because it contains a substantial quantity of silica and its ingesting leads to the absorption and use of calcium and the formation of collagen [92]. Besides, it contains several secondary metabolites that help prevent bone loss caused by age and estrogen deficiency [93]. Furthermore, the osteoblasts' proliferative effects of *E. arvense* together with its inhibitory action in osteoclasts functions have also been described [38, 94]. Corroborating these results, Kotwal and Badole [95] confirmed the beneficial effects of an extract of *E. arvense* on the bone mineral density of rats submitted to ovariectomy. Finally, Arbabzadegan et al. [96] also confirmed these *E. arvense* extract effects by analyzing the mandibular bone mineral density from rats.

*E. giganteum* has been traditionally used as an antidiabetic herbal remedy to treat diabetes. Recently, Vieira et al. [97] investigated the antidiabetic effects of the butanolic and aqueous extracts from *E. giganteum* in an animal model of diabetes. Both aqueous and butanolic extracts could significantly reduce glucose, cholesterol, and triacylglycerol, thus demonstrating their hypolipidemic and hypoglycemic effects. *E. giganteum* has also been intensively studied with the aim of preventing oral diseases because some studies showed its antimicrobial activity [10, 28, 98]. Also, despite the use of *E. giganteum* as a diuretic medicinal plant, Farinon et al. [9] evaluated the effect of its aqueous extract as immunomodulatory therapy in a model of antigen-induced arthritis (AIA). The treatment with the extract reduced nociception at 3, 6, and 24 h, decreased leukocyte migration, and inhibited lymphocyte proliferation. In conclusion, it was confirmed that *E. giganteum* extract has an anti-inflammatory and immunomodulatory potential in the acute inflammation model.

Studies in streptozotocin-induced diabetic rats showed that water and butanol extracts of the aerial parts of *E. myriochaetum* have significant hypoglycemic activity in an animal model [66]. Revilla et al. [99] also described the hypoglycemic effect of *E. myriochaetum* through a clinical trial. Type 2 diagnosed diabetic patients were analyzed and received a quantity of 0.33 g/kg of dried plant (aerial parts). This single dose of the extract significantly reduced the patients' blood glucose levels, proving a promising potential of this species in the therapeutic management of diabetic individuals.

Yeganegi et al. [40] evaluated *E. telmateia* extracts' antimicrobial activity against *Staphylococcus aureus*, *Bacillus cereus*, *Escherichia coli*, *Salmonella typhi*, and *Candida albicans*. The results of this group of authors showed that the extract of *E. telmateia* exhibited the highest antimicrobial potency against *S. aureus*. Besides, regarding the antimicrobial potential of another species, *E. hyemale*, two articles were found. The first was contributed by Queiroz et al. [61], who showed an antifungal activity of the extract against dermatophyte fungi (*Trichophyton rubrum* and *Microsporum canis*). The second, published by Alves et al. [100], showed the antibiofilm, antimicrobial, and antiparasitic potential of *E. hyemale* extract against several infectious agents (bacteria, fungi, *Mycobacterium*, and *Trypanosomes*).

All the pharmacological activities described here can benefit in parallel the therapeutic management of kidney disorders. The anti-inflammatory activity related to some species could mitigate the damage caused by the high blood pressure levels suffered by the kidneys during systemic arterial hypertension or even the damage caused by diabetes. Accordingly, some species have also demonstrated significant antidiabetic actions. In addition, regarding the antimicrobial actions described by some species, these could have a significantly useful application against genitourinary infections, which could also benefit kidney damage. The multiple therapeutic possibilities for plants of the *Equisetum* genus are evident; however, many studies are needed to evaluate both the pharmacological potential and the safety concerning the use of the preparations. Finally, Table 3 summarizes the main scientific findings that demonstrate the therapeutic effects of different species of *Equisetum* in kidney disorders and other biological activities.

## 6. Final Considerations

This review reveals that the most widespread traditional use of *Equisetum* is as diuretic, followed by the treatment of genitourinary diseases. The most popular species from the *Equisetum* genus with medicinal use is *E. arvense*. Other species also demonstrate beneficial effects in experimental assays for different pharmacological purposes. However, few *in vivo* toxicity studies were conducted so far for most species, so there is no consensus on effective and toxic dosage. Hence, many species need to be explored in detail for scientific validation of popular use to induce diuresis and treat kidney and other associated diseases.

## Figures and Tables

**Table 1:** Traditional applications of *Equisetum* genus stratified by country, species, plant part, and medicinal use.

Country	Species	Plant part	Preparation method	Medicinal use	Reference
Bolivia	*Equisetum giganteum L*.	Stem, entire plant	Decoction	Diarrhea, stomach heat, liver and kidneys diseases, inflammation	[8]
Brazil	*Equisetum giganteum L*.	Stems^a^, aerial parts^b^	Decoction, infusion^a^	Rheumatic diseases^a^ Diuretic, urinary disorders, inflammation^b^ Weight loss^c^	[9]^a^ [10]^b^ [11]^c^
	*Equisetum arvense* L.	Aerial parts	Infusion^a^	Diuretic^ab^ Remineralization, inflammation^b^	[12]^a^ [13]^b^
Mexico	*Equisetum myriochaetum* Schlecht and Cham	Aerial parts	Infusion^ab^	Diabetes type 2^abc^ Kidney diseases, diabetes^d^	[14]^a^ [15]^b^ [16]^c^ [17]^d^
Chile	*Equisetum bogotense* Kunth *Equisetum giganteum L*.	Aerial parts	Infusion	Diuretic	[18]
Lebanon	*Equisetum arvense* L*. Equisetum maximum* Lam.*Equisetum telmateia* Ehrh.	Aerial parts	Decoction	Antirheumatic, antineuralgic	[19]
Romania	*Equisetum arvense* L.	Sterile aerial, stems	Unreported	Inflammation^ab^ Ulcers, skin tumors, itching, wound healing, bruise, chilblains, leukorrhea, paronychia, foot hyperhidrosis, impetigo, furuncle, dermatitis, neurodermatitis^b^	[20]^a^ [21]^b^
Germany	*Equisetum arvense* L.	Aerial parts	Unreported	Inflammation	[22]
Serbia	*Equisetum arvense* L.	Whole plant^a^, sterile stems^b^	Infusion	Urogenital diseases, diuretic^a^ Kidney diseases, arthritis, bleeding ulcers, tuberculosis, wounds healing^b^	[23]^a^ [24]^b^
China	*Equisetum arvense* L. *Equisetum hyemale* L. *Equisetum ramosissimum* Desf.	Aerial parts Aerial parts Aerial parts	Unreported Unreported Unreported	Hemorrhage, urethritis, jaundice, hepatitis Hypertension, inflammation, acute stroke, bleeding, cancer Hemorrhage, urethritis, jaundice, hepatitis	[25–27]
Peru	*Equisetum giganteum* L.	Aerial parts	Unreported	Diarrhea, diuretic, emmenagogue, wound healing	[28]
Colombia	*Equisetum bogotense* Kunth	Whole plant	Infusion, decoction	Diuretic, kidney stones	[29]
India	*Equisetum arvense* L.	Aerial parts	Infusion	Strengthen bones, hair, nails	[30]
Italy	*Equisetum arvense* L.	Dried sterile stems	Infusion	Remineralizing, diuretic, inflammation, rheumatoid arthritis	[31]
Croatia	*Equisetum arvense* L.	Aerial parts	Infusion	Diabetes	[32]
Greece	*Equisetum arvense* L.	Aerial parts	Decoction	Urogenital disorders, prostatitis, diuretic, musculoskeletal diseases	[33]
Turkey	*Equisetum telmateia* Ehrh. *Equisetum palustre* L.	Aerial parts Aerial parts	Decoction Infusion	Acne, rheumatism, broken bones, diuretic, expectorant, kidney stones, strengthening hair, skin, and nails Peptic ulcer, hemorrhoids, kidney stones	[34, 35]
Portugal	*Equisetum telmateia* Ehrh. *Equisetum arvense L*.	Aerial parts Aerial parts	Unreported Unreported	Prostatitis, stomachaches, and cystitis^a^ Urinary, kidney, gastrointestinal disorders^b^ Arthritis, kidney diseases, bleeding ulcers, hepatitis, jaundice, and tuberculosis	[36]^a^ [37]^b^ [38]
Iran	*Equisetum arvense* L. *Equisetum telmateia* Ehrh.	Aerial parts Aerial parts	Unreported Unreported	Wound healing, strengthening of the bones, teeth, nails, and hair, gout, nosebleeds, urinary and prostate disorders, menorrhagia, rheumatoid arthritis Prostatitis, stomachache, inflammation, diarrhea, mouth infections, chronic eczema, antifungal	[39, 40]
Thailand	*Equisetum arvense* L. *Equisetum debile* Roxb. ex Vaucher	Aerial parts Aerial parts	Unreported Unreported	Hair loss Diuretic, wound healing, muscle relaxant, hair growth stimulant, hair loss	[41]
Spain	*Equisetum telmateia* Ehrh. *Equisetum arvense* L. *Equisetum ramosissimum* Desf.	Aerial parts Aerial parts Aerial parts	Infusion^ab^ Decoction, infusion^ab^ Infusion^a^	Hypertension, diuretic^a^ Bones disorders, to strengthen broken bones^b^ Hypertension, diuretic^a^ Bone disorders, to strengthen broken bones^b^ Depurative, diuretic^a^	[1]^a^ [42]^b^

Equal letters in the same line relate plant part, preparation method, and medicinal use to respective studies (column of references).

**Table 2:** Phytochemistry of *Equisetum* genus stratified by species, plant part, method, and compounds.

Species	Plant part	Extract/method	Compounds	Reference
*Equisetum arvense* L.	Rhizomes	MeOH/CC-HPLC	3′-Deoxyequisetumpyrone	[51]
			4′-*O*-Methylequisetumpyrone	
	Aerial parts	EtOAc fraction/CC	Onitin	[25]
			Onitin-9-*O*-glucoside	
			Apigenin	
			Luteolin	
			Kaempferol 3-*O*-glucoside	
			Quercetin 3-*O*-glucoside	
		EtOAc fraction/HPLC-DAD	Quercetin 3-*O*-glucoside	[52]
			Apigenin 5-*O*-glucoside	
			Kaempferol 3-*O*-glycoside	
		n-BuOH fraction/HPLC-DAD	Quercetin 3-*O*-glucoside	
			Di-*E*-caffeoyl-meso-tartaric acid	
		H_2_O fraction/HPLC-DAD	Di-*E*-caffeoyl-meso-tartaric acid	
			Phenolic acid 1	
			Phenolic acid 2	
		Petrol/PTLC-GC-MS	Taraxerol	[53]
			*β*-amyrin	
			Germanicol	
			*α*-Amyrin	
			Ursolic acid	
			Oleanolic acid	
			Betulinic acid	
			Isobauerenol	
			Epicholestanol	
			Cholesterol	
			Sitosterol	
			28-Isofucosterol	
			Taraxasterone	
			*ψ*-Taraxasterone	
	Unreported	Ethanol decoct/HPLC	Mono-, di-, and triglycosides of kaempferol	[22]
			Quercetin	
			Apigenin	
			Genkwanin	
			Protogenkwanin	
			Mono- and dicaffeoyl-tartaric acid	
	Fertile sprouts	Water-soluble/spectroscopic analysis	Equisetumoside A-C	[54]
			Uridine	
			Inosine	
			2′-Deoxyinosine	
			2′-Deoxycytidine	
			Tryptophan	
			Thymidine	
			5-Carboxy-2′-deoxyuridine	
			Coniferin	
			Kaempferol 3-*O*-*β*-D-sophoroside-7-*O*-*β*-D-glucopyranoside	
	Stems	Hydroalcoholic/HPLC	Quercetin	[24]
			Quercetin 3-*O*-glucoside	
			Quercetin 3-*O*-(6″-*O*-malonylglucoside)	
			5-*O-C*affeoyl shikimic acid	
			Monocaffeoyl meso-tartaric acid	
			Dicaffeoyl meso-tartaric acid	
	Aerial parts	Diethyl ether/GC-MS	Benzaldehyde	[55]
			Phenylethanal	
			2-Phenylethanol	
			4-Vinylguaiacol	
			Isovanillin	
			Homovanillic acid	
			2,3-Octanedione	
			Hexanoic acid	
			(*Z*)-3-Hexanoic acid	
			(*E*)-2-Hexanoic acid	
			Dihydroactinidiolide	
			(*E*,*Z*)-Pseudoionone	
			(*E*,*E*)-Pseudoionone	
			3-Hydroxy-*β*-ionol	
			4-Hydroxy-*β*-ionol	
			3-Oxo-*α*-ionol	
			3-Hydroxy-7,8-dihydro-*β*-ionol	
			3-Hydroxy-7,8-epoxy-*β*-ionol	
			4-Oxo-*β*-ionone	
			4-Hydroxy-7,8-dihydro-*β*-ionol	
*Equisetum bogotense* Kunth	Unreported	Acidic/HILIC HPLC	Nicotine	[56]
			Palustrine	
			Palustridiene	
*Equisetum debile* Roxb. ex Vaucher	Whole plant	EtOAc/CC	Phenylhexane debilitriol	[57]
			Equisetumine	
			Guaiacylglycerol-*β*-coniferyl ether	
			5-Hydroxymethyl-2-furfuraldehyde	
			Coumaric acid	
			*p*-Hydroxybenzoic acid	
			Ferulic acid	
		BuOH/CC	(+)-Lariciresinol 9-*O*-*β*-D-glucopyranoside	
			8-*O*-4′ Neolignan glucoside debilignanoside	
			Equisetumoside B	
			(+)-Isolariciresinol-3-*α*-*O*- *β*-D-glucopyranoside	
			Thymidine	
		EtOAc/CC	Blumenol A	[58]
			Corchoinoside C	
		BuOH/CC	Sammangaoside A	
			Debilosides A–C	
			(3S,5R,6R,7E,9S)‐9‐[(*β*‐D‐Glucopyranosyl)oxy]megastigm‐7‐ene‐3,5,6‐triol	
	Aerial parts	MeOH/CC	Macarangioside D	[59]
			Sammangaoside A	
			(3S,5R,6S,7E,9S)-Megastigman-7-ene-5,6-epoxy-3,9-diol 3,9-*O*- *ß*-D-diglucopyranoside	
			(6R,9S)-3-Oxo-*α*-ionol 9-*O*-*β*-D-glucopyranoside	
			Debiloside B	
			Kaempferol 3-*O*-sophoroside	
			Kaempferol 3,7-*O*-*β*-D-diglucopyranoside	
			Kaempferol 3-*O*-sophoroside-7-*O*- *ß*-D-glucopyranoside	
			Phenylethyl *O*- *ß*-D-glucopyranoside	
			(*Z*)-3-Hexanyl *O*-*β*-D-glucopyranoside	
			(7S,8R)-Dehydrodiconiferyl 4-*O*- *ß*-D-glucopyranoside	
			L-tryptophan	
*Equisetum giganteum L*.	Stems	Hydroethanolic/LC-DAD and LC–ESI-MS/MS	Quercetin tri-*O*-hexoside	[60]
			Kaempferol 3-*O*-sophoroside-7-*O*-glucoside	
			3-Hydroxyhispidin-3,4′-di-*O*-glucoside	
			Quercetin 3,7-di-*O*-glucoside	
			Quercetin 3-*O*-(caffeoyl)-glucoside	
			Kaempferol 3,7-di-*O*-glucoside	
			Flavonol-di-*O*-hexoside	
			Equisetumpyrone	
			Caffeic acid derivative	
			Kaempferol 3-*O*-sophoroside	
			Ferulic acid derivative	
			Kaempferol 3-*O*-glucoside	
	Aerial parts	Hydroethanolic/RP-UHPLC, UV-Vis, and MS/MS^n^	Kaempferol 3-*O*-sophoroside-7-*O*- glucoside	[10]
			3-Hydroxyhispidin-3,4′-di-*O*-glucoside	
			Quercetin 3,7-di-*O*-glucoside	
			Quercetin 3-*O*-(caffeoyl)-glucoside	
			Kaempferol 3,7-di-*O*-glucoside	
			Flavonol-di-*O*-hexoside	
			Equisetumpyrone	
			Caffeic acid derivative	
			Kaempferol 3-*O*-sophoroside	
			Quercetin-hexoside	
			Ferulic acid derivative	
			Kaempferol 3-*O*-glucoside	
	Unreported	Acidic/HILIC HPLC	Palustridiene	[56]
*Equisetum hyemale* L.	Aerial parts	Hydroalcoholic/HPLC–PAD	Gallic acid	[61]
			Tannic acid	
			Chlorogenic acid	
			Caffeic acid	
		n-BuOH/CC	Quercetin	[62]
			Kaempferol 7-*O*- *β*-D-glucopyranoside	
			*α*-D-fructofuranose	
			5-Hydroxymethylfurfural	
			L-Uridine	
			Kaempferol 3,7-di-*O*-*β*-D-glucopyranoside	
			Kaempferol 3-sophoroside-7-*O*-*β*-D-glucopyranoside	
			Kaempferol 7-*O*-*α*-L-rhamnoside-4′-*O*-*β*-D-glycopyranoside	
			2-(Sophorosyl)-1-(4-hydroxyphenyl) ethanone	
*Equisetum myriochaetum* Schlecht. and Cham.	Aerial parts	H_2_O and BuOH/CC-HPLC	Kaempferol 3-*O*-sophoroside	[63]
			Kaempferol 3,7-di-*O*-*β*-glucoside	
			Kaempferol 3-*O*-sophoroside-4′-*O*- *β*-glucoside	
			Caffeoyl-methylate-4- *β*-glucopyranoside	
	Unreported	Unreported	Chrysin	[64]
			*β*-Sitosterol	
			*β*-D-glucosylsitosterol	
			*β*-D-glucose	
			Fatty acids	
*Equisetum palustre* L.	Unreported	Acidic/HILIC HPLC	Nicotine	[56]
			Palustrine	
			Palustridiene	
	Whole plant	Acidic fraction/HPLC–ESI-MS/MS	Palustrine	[50]
			N^5^-Formylpalustrine	
			N^5^-Acetylpalustrine	
			18-Deoxypalustrine	
			Palustridiene	
			N^5^-Formylpalustridiene	
			Myricoidine	
			Spermidine	
	Aerial parts	n-BuOH/HS-CCCC	Kaempferol 3-*O*-1”- *β*-D-glucopyranosyl-3-*O*-1‴- *β* -D-glucopyranoside	[35]
	Unreported	MSPD extraction/UHPLC–MS/MS	Kaempferol 3-O-1”- *β*-D-glucopyranosyl-3-O-1‴- *β* -D-glucopyranoside	[65]
			4-*O*-(*p*-coumaroyl)shikimic acid	
			Luteolin-7-*O*- *β*-D-glucopyranoside	
			Quercetin-3-*O*- *β*-D-glucopyranoside	
			Apigenin-5-*O*- *β*-D-glucopyranoside	
			Genkwanin-5-*O*- *β*-D-glucopyranoside	
			Luteolin	
			Apigenin	
			Genkwanin	
	Stems	Hydroalcoholic/HPLC	Kaempferol	[24]
			Kaempferol 3-*O*-glucoside	
			Kaempferol 3′-*O*-rutinoside	
			Kaempferol 3*-O*-rutinoside-7-*O*-sophoroside	
			Kaempferol 3-*O*-rutinoside-7-*O*-glucoside	
			Monocaffeoyl meso-tartaric acid	
	Aerial parts	Diethyl ether/GC-MS	Benzaldehyde	[55]
			Phenylethanal	
			2-Phenylethanol	
			4-Vinylguaiacol	
			Isovanillin	
			Homovanillic acid	
			2,3-Octanedione	
			Hexanoic acid	
			(*Z*)-3-Hexanoic acid	
			(*E*)-2-Hexanoic acid	
			Dihydroactinidiolide	
			(*E,Z*)-Pseudoionone	
			(*E,E*)-Pseudoionone	
			3-Hydroxy-*β*-ionol	
			4-Hydroxy-*β*-ionol	
			3-Oxo-*α*-ionol	
			3-Hydroxy-7,8-dihydro-*β*-ionol	
			3-Hydroxy-7,8-epoxy-*β*-ionol	
			4-Oxo-*β*-ionone	
			4-Hydroxy-7,8-dihydro-*β*-ionol	
*Equisetum ramosissimum* Desf.	Aerial parts	Diethyl ether/GC-MS	Phenylethanal	[55]
			2-Phenylethanol	
			Phenylacetic acid	
			4-Vinylguaiacol	
			(*Z*)-Ferulic acid	
			(*E*)-Ferulic acid	
			(*E*)-2-Heptenal	
			Hexanoic acid	
			Heptanoic acid	
			(*E*)-2-decenal	
			Nonanoic acid	
			(*E,Z*)-2,4-Decadienal	
			(*E,E*)-2,4-Decadienal	
			Lauric acid	
			3-Hydroxy-7,8-epoxy-*β*-ionol	
*Equisetum telmateia* Ehrh.	Stems	Hydroalcoholic/HPLC	Kaempferol	[24]
			Kaempferol 3-*O*-glucoside	
			Kaempferol 3-*O*-(6″-O-acetylglucoside)	
			Kaempferol 3,7-*O*-diglucoside	
			Kaempferol 3-*O*-(6″-*O*-acetylglucoside)-7-*O*-glucoside	
			Kaempferol 3-*O*-glucoside-7-*O*-rhamnoside	
			Kaempferol 3-*O*-(6″-*O*-acetylglucoside)-7-*O*-rhamnoside	
			Kaempferol 3-*O*-rutinoside-7-*O*-glucoside	
			5-*O*-Caffeoyl shikimic acid	
			Monocaffeoyl meso-tartaric acid	
	Aerial parts	EtOAc fraction/HPLC-PAD-ESI/MS	Protocatechuic acid	[36]
			*p*-Hydroxybenzoic acid	
			Proanthocyanidin tetramer containing an Afz residue	
			Procyanidin dimer B2	
			Caffeic acid derivative	
			A-type proanthocyanidin trimer	
			Caffeic acid derivative	
			Procyanidin trimer C1	
			Proanthocyanidin trimer (epi)Afz-(epi)C-(epi)C	
			Kaempferol acetyl-dihexose	
			Proanthocyanidin trimer (epi)C-(epi)C-(epi)Afz	
			Proanthocyanidin tetramer containing two Afz residues	
			Kaempferol glucoside-rhamnoside	
			Caffeic acid derivative	
			Kaempferol acetylglucoside-rhamnoside	
			Kaempferol 3-*O*-glucoside	
			Kaempferol 3- *O*-acetylglucoside	
	Aerial parts		Benzyl alcohol	[55]
			Phenylethanal	
			4-Vinylguaiacol	
			Isovanillin	
			2,3-Octanedione	
			(*Z*)-3-Hexanoic acid	
			*β*-Caryophyllene	
			*α*-Ionone	
			(*E,E*)-Pseudoionone	
			3-Oxo-7,8-dihydro-*α*-ionone	
			3-Hydroxy-*α*-ionone	
			3-Oxo-*α*-ionol	
			3-Hydroxy-7,8-epoxy-*β*-ionol	
			4-Hydroxy-7,8-dihydro-*β*-ionol	

Afzelechin (Afz); column chromatography (CC); gas chromatography (GC); epicatechin or catechin ((epi)C); epiafzelechin or afzelechin ((epi)Afz); high-performance liquid chromatography (HPLC); high-speed centrifugal countercurrent chromatography (HS-CCCC); diode-array detector (DAD); hydrophilic interaction chromatography (HILIC); liquid chromatography (LC); electrospray ionization (ESI); mass spectrometry (MS); reversed-phase ultra-high-performance liquid chromatography (RP-UHPLC); pulsed amperometric detection (PAD); ultraviolet–visible spectroscopy (UV-Vis); preparative thin layer chromatography (PTLC).

**Table 3:** Pharmacological properties of *Equisetum* genus stratified by effect, key findings, and species.

Effect	Key findings	Species	Reference
Diuretic activity	Increased excretion of sodium, potassium, and chloride	*Equisetum fluviatile*	[75]
		*Equisetum hyemale* var. *affine*	
		*Equisetum giganteum*	
		*Equisetum myriochaetum*	
	Increased excretion of sodium, potassium, and chloride^*∗*^	*Equisetum bogotense*	[18]
	No effects on the urinary excretion of electrolytes^*∗*^	*Equisetum arvense L*.	[13]
Urinary bladder disorders	Increased interval between urinary bladder contractions and decreased bladder contraction pressure	*Equisetum arvense L*.	[67]
Urinary incontinence	Improvement in urinary incontinence episodes; day-time urination frequency; and gain in nocturnal urinary frequency^*∗*^	CELcomplex® (*Cucurbita pepo* L*.; Equisetum arvense* L.; and *Linum usitatissimum* L.)	[68]
State of hydration and bloating sensation	Decreased extracellular water and fat mass^*∗*^	Formula (*Fraxinus ornus* L.; *Ananas comosus* L.; *Betula pendula* R.; *Equisetum arvense* L.; *Urtica dioica L*; and *Pilosella officinarum* L. Vaill.)	[69]
Nephrolithiasis	Lower calcium oxalate crystals	Formula (*Herniaria glabra* L.; *Agropyron repens*; *Equisetum arvense* L.; and *Sambucus nigra L*.)	[70]
Antinociceptive effect	Inhibitory action on chemically induced acute pain test	*Equisetum arvense L*.	[84]
	Reduction of clinical chronic musculoskeletal pain^*∗*^	*Equisetum arvense L*.	[86]
Anti-inflammatory effect	Anti-inflammatory action on COPD model	*Equisetum arvense L*.	[85]
	Reduction of antigen-induced arthritis model	*Equisetum giganteum L*.	[9]
Cytotoxic and anticancer effect	Reduction in cell viability of cancer cells	*Equisetum arvense L*.	[87–90]
Effect on bone diseases	Increase in calcium absorption	*Equisetum arvense L*.	[92]
	Proliferation of osteoblasts or inhibitory action in osteoclasts	*Equisetum arvense L*.	[38, 94]
	Bone resorption markers of rats	*Equisetum arvense L*.	[95]
	Bone mineral density of rats	*Equisetum arvense L*.	[95, 96]
Antidiabetic	Beneficial effects in streptozotocin-induced diabetic rats	*Equisetum myriochaetum* Schlecht. and Cham.	[66]
	Clinically beneficial to recently diagnosed type 2 diabetic patients^*∗*^	*Equisetum myriochaetum* Schlecht. and Cham.	[99]
	Beneficial effects in alloxan-induced diabetic rabbits	*Equisetum giganteum L*.	[97]
	Reduction in oxidative stress	*Equisetum arvense L*.	[101]
Antioxidant and antimicrobial	Antimicrobial activity	*Equisetum giganteum L*.	[10, 28, 98]
	Antioxidant and antimicrobial of supercritical fluid extraction	*Equisetum telmateia* Ehrh.	[40]
	Bactericidal, fungicidal, and antiprotozoal effect	*Equisetum hyemale L*.	[61, 100]
	Bactericidal and fungicidal activity	*Equisetum arvense L*.	[91]
	Radical scavenger activity	*Equisetum arvense L*.	[102]
		*Equisetum ramosissimum* Desf. and *Equisetum telmateia* Ehrh.	
	Radical scavenger activity and antimicrobial activity	*Equisetum arvense L*.	[24]
		*Equisetum sylvaticum L*.	
		*Equisetum fluviatile L*.	
		*Equisetum palustre L*.	
		*Equisetum telmateia* Ehrh.	
	Radical scavenger activity and antimicrobial activity	*Equisetum telmateia* Ehrh.	[47]
Effects on central nervous system	Anticonvulsant and sedative effects	*Equisetum arvense L*.	[103]
	Prolonged the ketamine-induced total sleeping time and decreased the locomotor activity in mice	*Equisetum arvense L*.	[104]
Antiulcer	Gastroprotective potential against different ulcerogenic agents	*Equisetum palustre L*.	[35]

^*∗*^Clinical study.

## Supplementary Materials

Supplementary Materials Figure S1: chemical structure of the compounds identified in the *Equisetum* genus. Chemical structures are presented followed by their names, molecular weights, and references.
