# Supplementary material for: Phytochemistry and Pharmacology of the Genus Equisetum (Equisetaceae): A Narrative Review of the Species with Therapeutic Potential for Kidney Diseases
Source: Evid Based Complement Alternat Med. 2021 Mar 5;2021:6658434. doi: 10.1155/2021/6658434 (PMC7954623; doi:10.1155/2021/6658434)
Supplement: Supplementary Materials — Figure S1: chemical structure of the compounds identified in the Equisetum genus. Chemical structures are presented followed by their names, molecular weights, and references. [file 6658434.f1.docx]

**SUPPLEMENTARY MATERIAL**

**Phytochemistry and pharmacology of the genus *Equisetum* (*Equisetaceae*): A narrative review of the species with therapeutic potential for kidney diseases**

Thaise Boeing^1^, Karyne Garcia Tafarelo Moreno^2^; Arquimedes Gasparotto Junior^2^, Luisa Mota da Silva^3^, Priscila de Souza^3*^

^1^Escola de Ciências Farmacêuticas de Ribeirão Preto, Universidade de São Paulo, Ribeirão Preto, São Paulo, Brazil.

^2^Laboratório de Farmacologia Cardiovascular- LaFaC, Faculdade de Ciências da Saúde, Universidade Federal da Grande Dourados, Dourados, MS, Brazil.

^3^Programa de Pós-graduação em Ciências Farmacêuticas, Núcleo de Investigações Químico-Farmacêuticas, Universidade do Vale do Itajaí, Itajaí, Brazil.

*Corresponding author:

E-mail address: tize.thaise@gmail.com (T. Boeing).

Escola de Ciências Farmacêuticas de Ribeirão Preto, Universidade de São Paulo, Avenida do Café S/N, 14040-903, Ribeirão Preto, São Paulo, Brazil.

Telephone number: +55 047 984296185

**FIGURE S1**

| 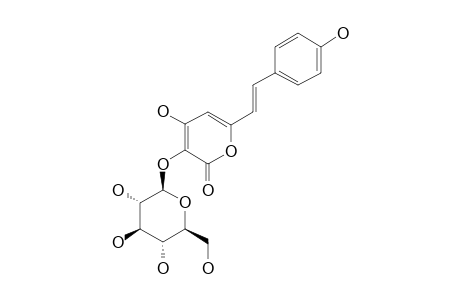  (1)  3'-Deoxyequisetumpyrone  MW: 408.3 g/mol  [105] | 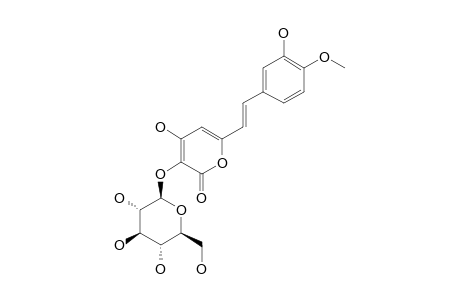  (2)  4'-*O*-Methylequisetumpyrone  MW: 438.3 g/mol  [105] | 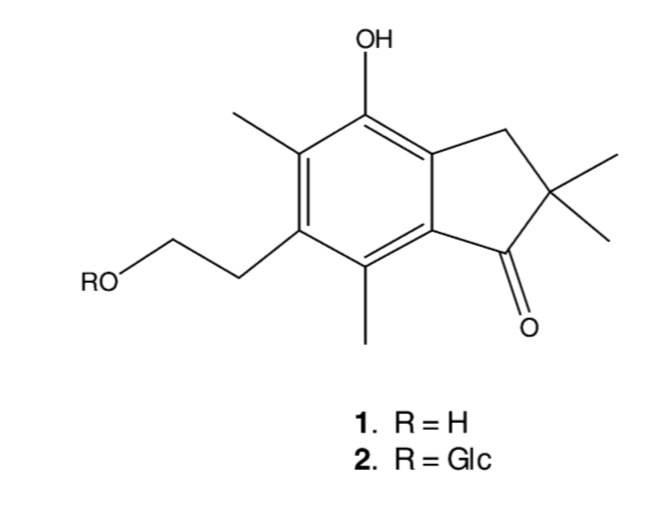  (3)  Onitin (R = H)  MW: 248.32 g/mol  (4)  Onitin-9-*O*-glucoside (R = Glc)  MW: 410.5 g/mol  [16] |
| --- | --- | --- |
| 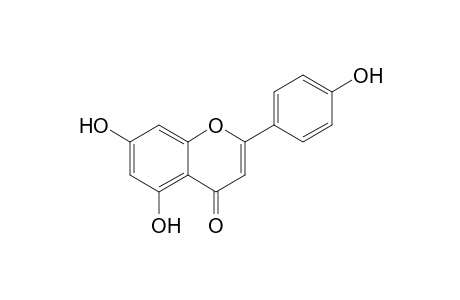  (5)  Apigenin  MW: 270.2 g/mol  [105] | 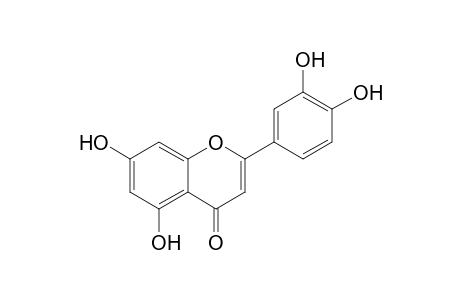  **(6)**  Luteolin  MW: 286.2 g/mol  [105] | 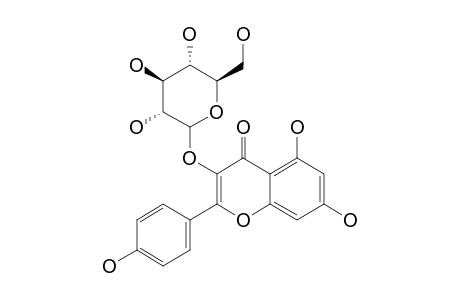  **(7)**  Kaempferol 3-*O*-Glucoside  MW: 448.3 g/mol  [105] |
| 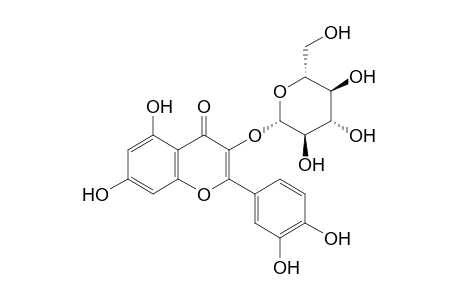  (8)  Quercetin 3-*O*-Glucoside, SYN: Isoquercitrin  MW: 464.3 g/mol  [105] | 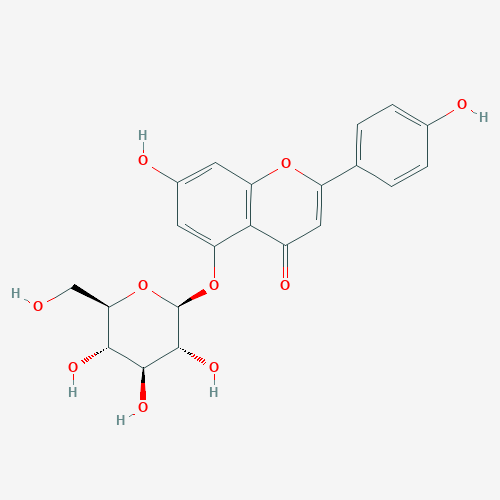  **(9)**  Apigenin 5-*O*-Glucoside  MW: 432.4 g/mol  [106] | 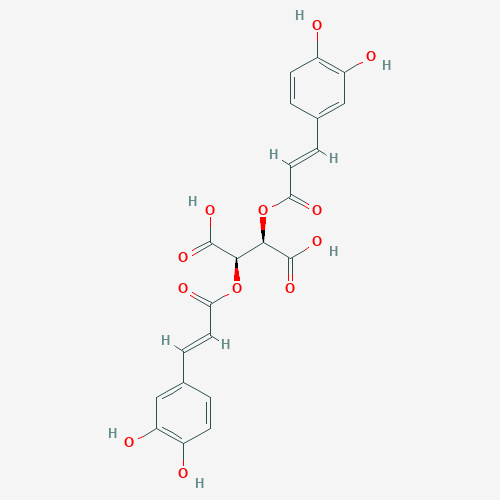  **(10)**  Di-E-Caffeoyl-meso-tartaric acid  MW: 474.3 g/mol  [106] |
| 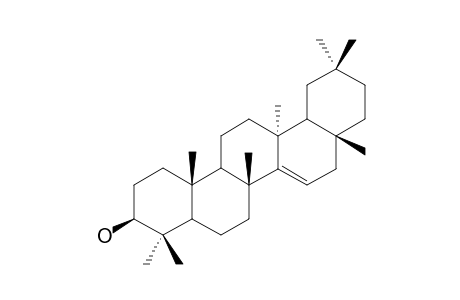  (11)  Taraxerol  MW: 426.7 g/mol  [105] | 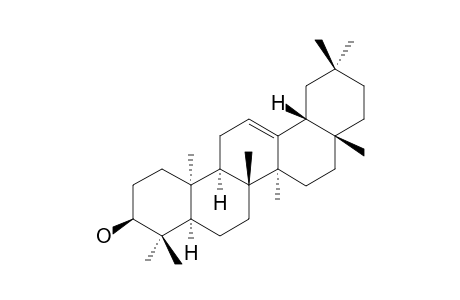  **(12)**  β-Amyrin  MW: 426.7 g/mol  [105] | 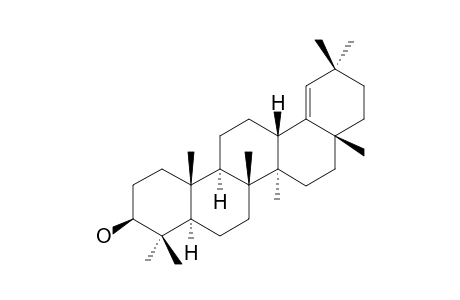  **(13)**  Germanicol  MW: 426.7 g/mol  [105] |
| 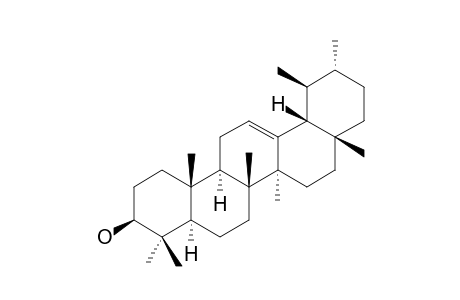(14)  α-Amyrin  MW: 426.7 g/mol  [105] | 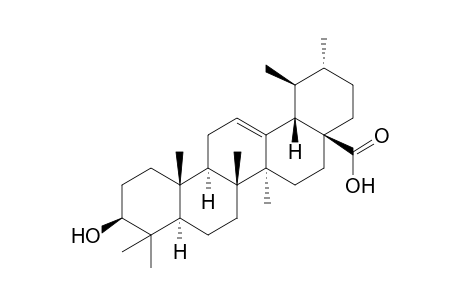  **(15)**  Ursolic acid  MW: 456.7 g/mol  [105] | 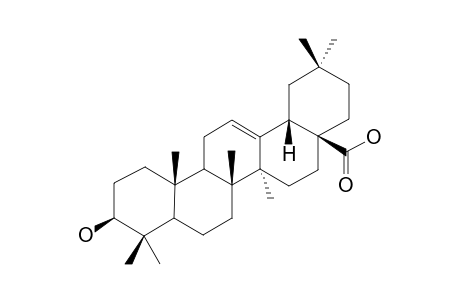  **(16)**  Oleanolic acid  MW: 456.7 g/mol  [105] |
| 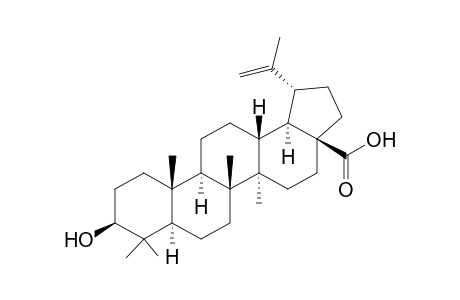  (17)  Betulinic acid  MW: 456.7 g/mol  [105] | 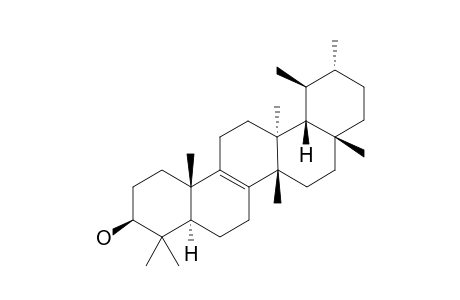  **(18)**  Isobauerenol  MW: 426.7 g/mol  [105] | 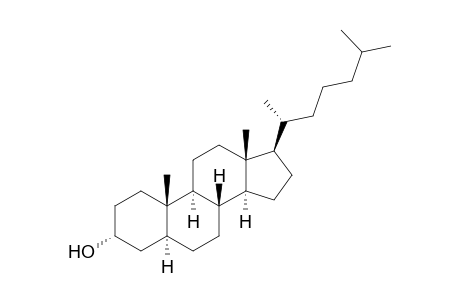  **(19)**  Epicholestanol  MW: 388.7 g/mol  [105] |
| 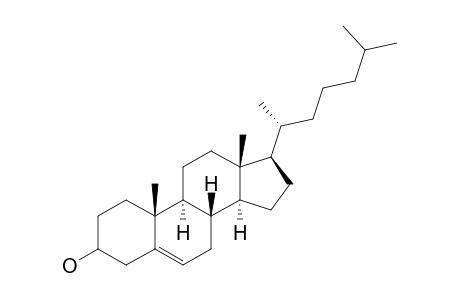  (20)  Cholesterol  MW: 386.7 g/mol  [105] | 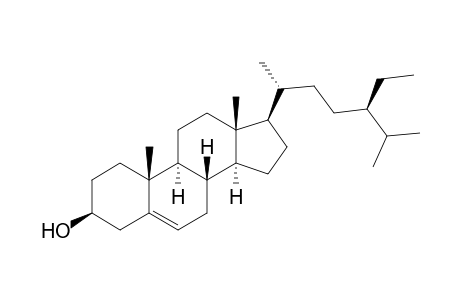  **(21)**  Sitosterol  MW: 414.7 g/mol  [105] | 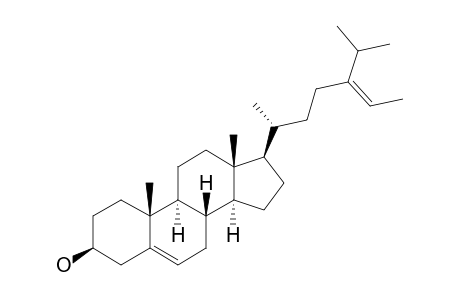  **(22)**  28-Isofucosterol  MW: 412.7 g/mol  [105] |
| 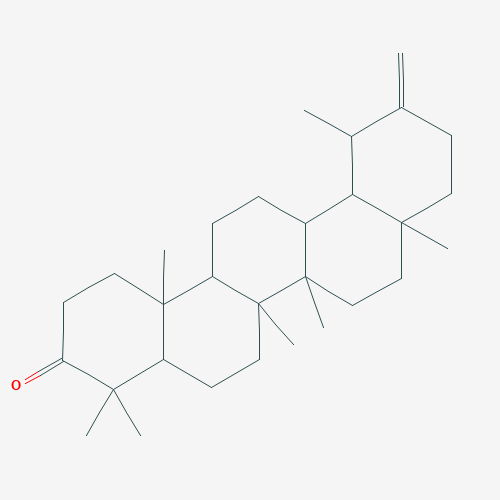  (23)  Taraxasteron  MW: 424.7 g/mol  [106] | 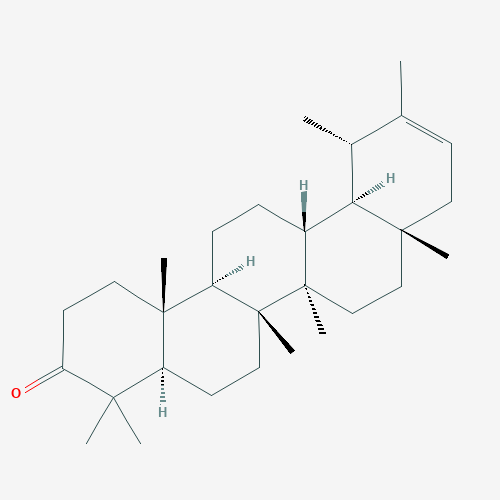  **(24)**  ψ-Taraxasterone  MW: 424.7 g/mol  [106] | 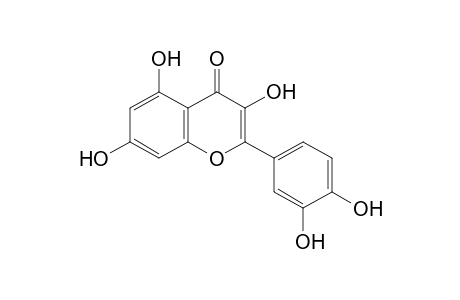  **(25)**  Quercetin  MW: 302.2 g/mol  [105] |
| 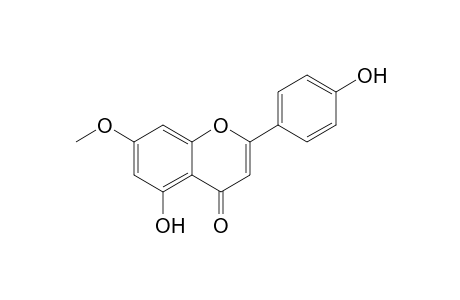  (26)  Genkwanin  MW: 284.2 g/mol  [105] | 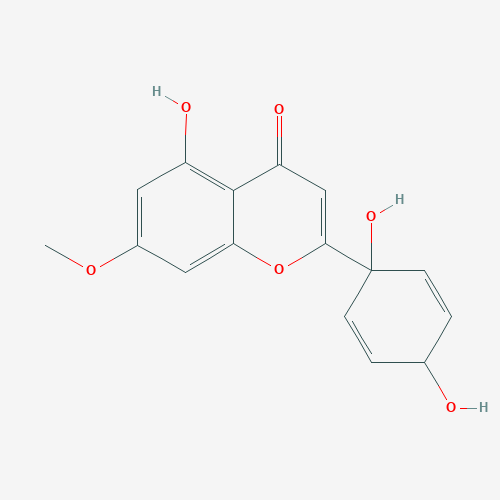  **(27)**  Protogenkwanin  MW: 302.2 g/mol  [106] | 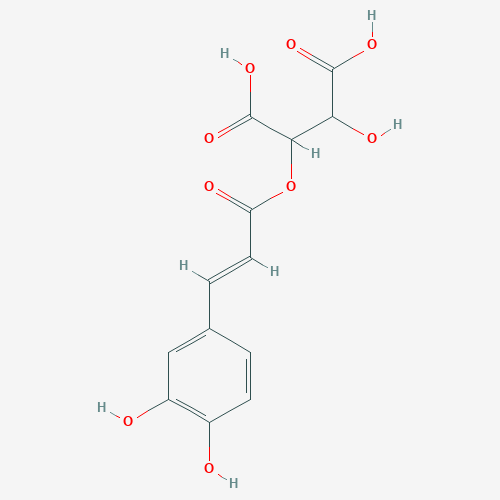  **(28)**  Monocaffeoyl-tartaric acid  MW: 312.2 g/mol  [106] |
| 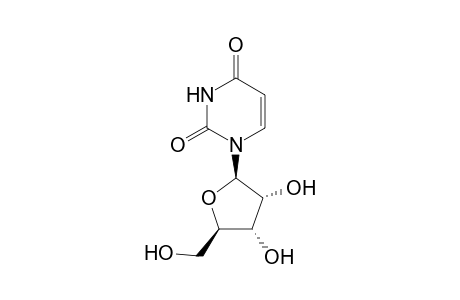  (29)  Uridine  MW: 244.2 g/mol  [105] | 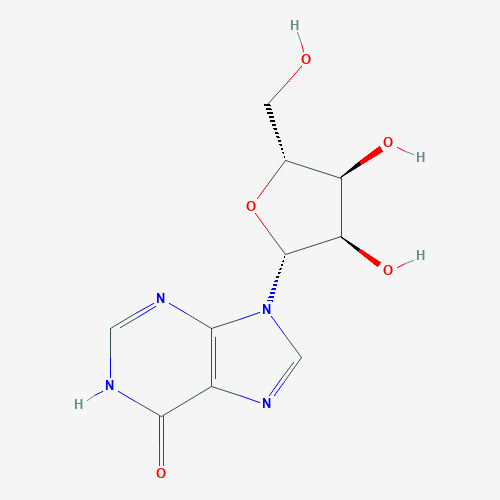  **(30)**  Inosine  MW: 268.2 g/mol  [106] | 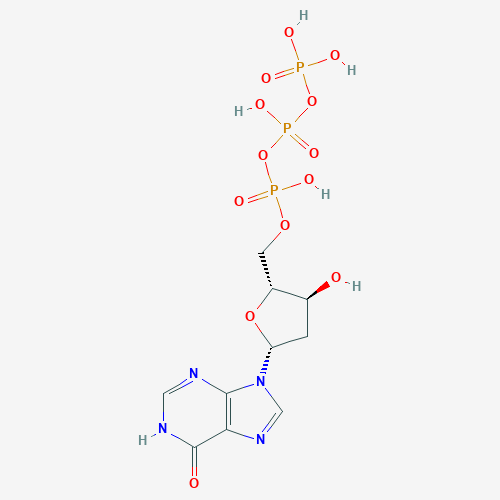  **(31)**  2′-Deoxyinosine  MW: 492.1 g/mol  [106] |
| 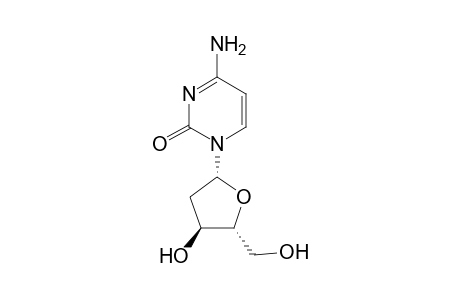  (32)  2′-deoxycytidine  MW: 227.2 g/mol  [105] | 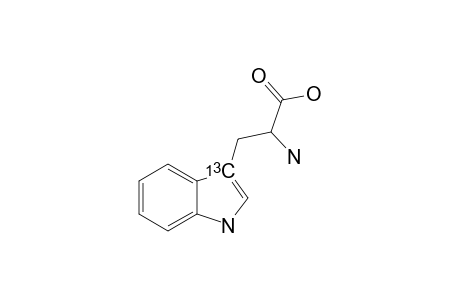  **(33)**  Tryptophan  MW: 205.2 g/mol  [105] | 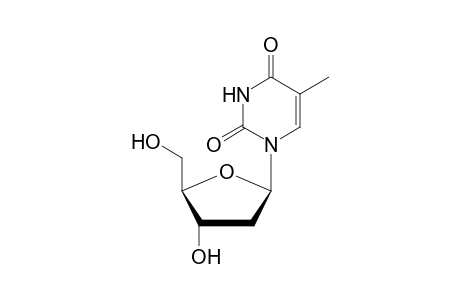  **(34)**  Thymidine  MW: 242.2 g/mol  [105] |
| 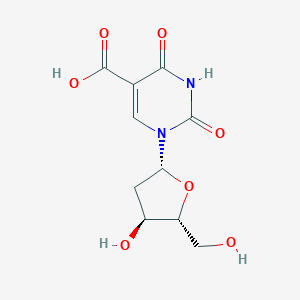  (35)  5-carboxy-2′-deoxyuridine  MW: 272.2 g/mol  [106] | 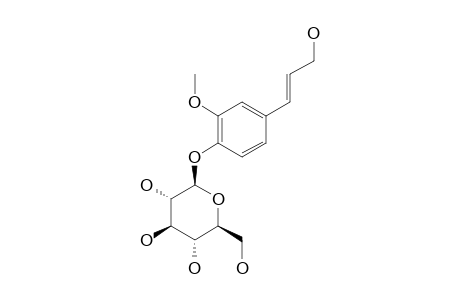  **(36)**  Coniferin  MW: 342.3 g/mol  [105] | 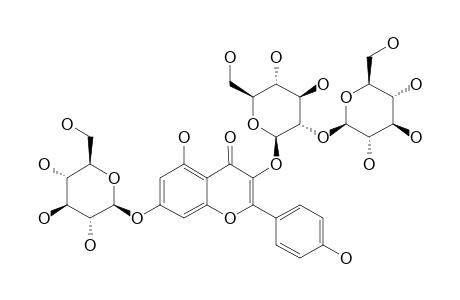  **(37)**  Kaempferol 3-*O* -β-D-sophoroside-7-*O*-β-D-glucopyranoside  MW: 772.7 g/mol  [105] |
| 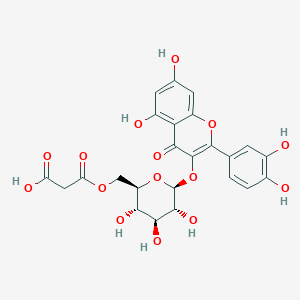  (38)  Quercetin 3-*O*-(6’’-*O*-malonylglucoside)  MW: 550.4 g/mol  [106] | 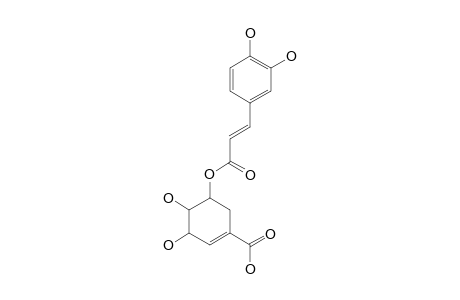  **(39)**  5-*O*-caffeoyl shikimic acid  MW: 336.3 g/mol  [105] | 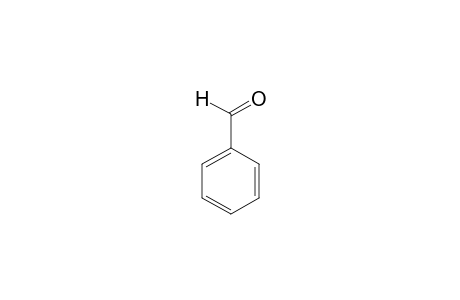  **(40)**  Benzaldehyde  MW: 106.1 g/mol  [105] |
| 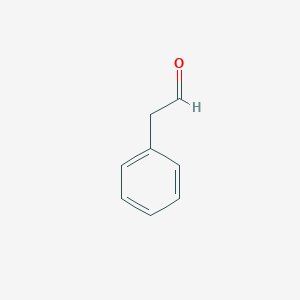  (41)  Phenylethanal  MW: 120.1 g/mol  [106] | 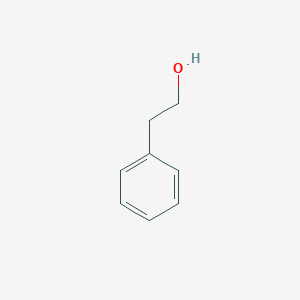  **(42)**  2-phenylethanol  MW:122.1  [106] | 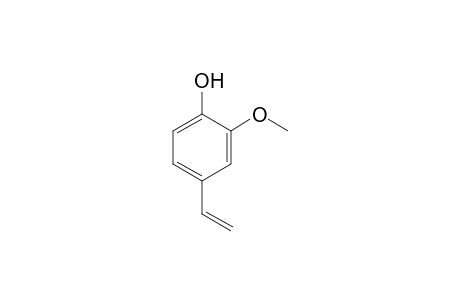  **(43)**  4-vinylguaiacol  MW: 150.1 g/mol  [105] |
| 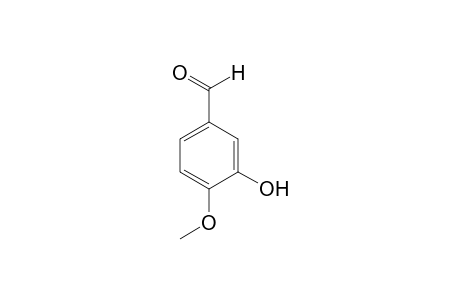  (44)  Isovanillin  MW: 152.1 g/mol  [105] | 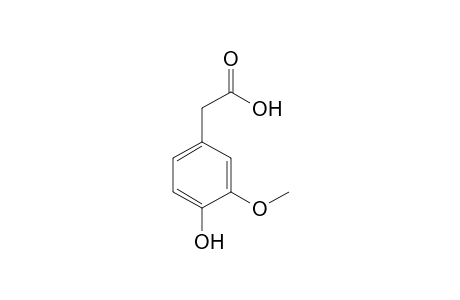  **(45)**  Homovanillic acid  MW: 182.1 g/mol  [105] | 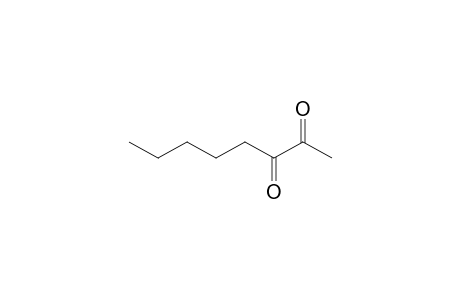  **(46)**  2,3-Octanedione  MW: 142.2 g/mol  [105] |
| 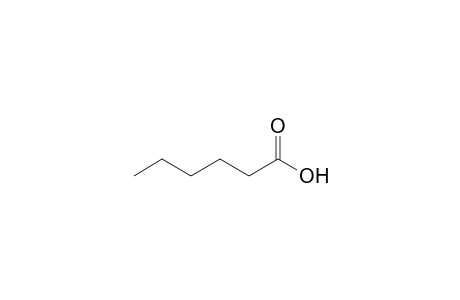  (47)  Hexanoic acid  MW: 116.1 g/mol  [105] | 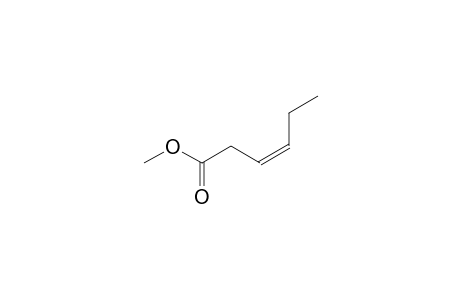  **(48)**  (Z)-3-hexenoic acid  MW: 128.1 g/mol  [105] | 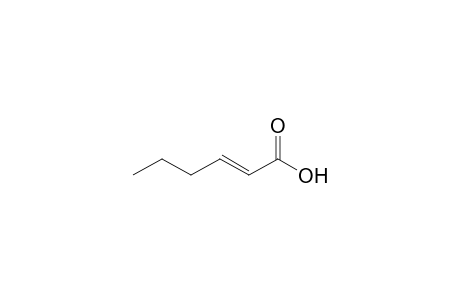  **(49)**  (E)-2-hexenoic acid  MW: 114.1 g/mol  [105] |
| 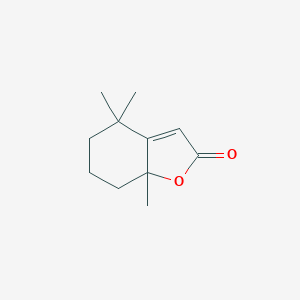  (50)  Dihydroactinidiolide  MW: 180.2 g/mol  [106] | 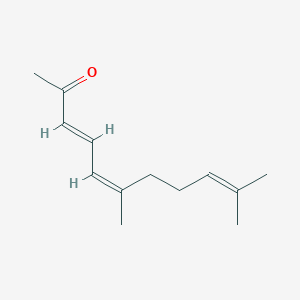  **(51)**  Pseudoionone  MW: 192.3 g/mol  [106] | 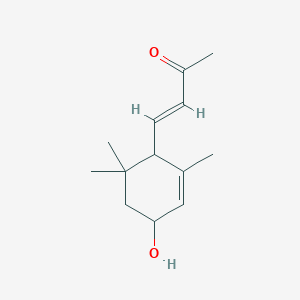  **(52)**  3-hydroxy-α-ionone  MW: 208.3 g/mol  [106] |
| 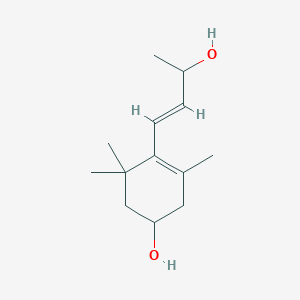  (53)  3-hydroxy-β-ionol  MW: 210.3 g/mol  [106] | 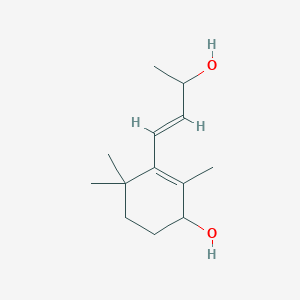  **(54)**  4-hydroxy-β-ionol  MW: 210.3 g/mol  [106] | 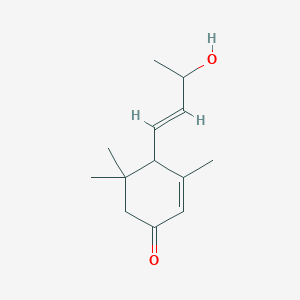  **(55)**  3-oxo-α-ionol  MW: 208.3 g/mol  [106] |
| 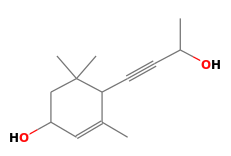  (56)  3-hydroxy-7,8-dihydro-β-ionol  MW: 208.2 g/mol  [110] | 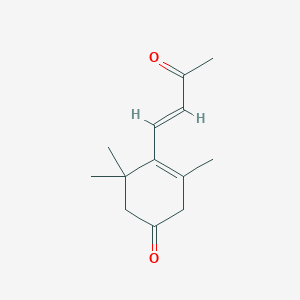  **(57)**  4-oxo-β-ionone  MW: 206.2 g/mol  [106] | 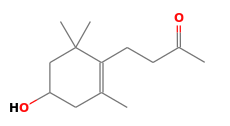  **(58)**  4-hydroxy-7,8-dihydro- β -ionone  MW: 210.3 g/mol  [110] |
| 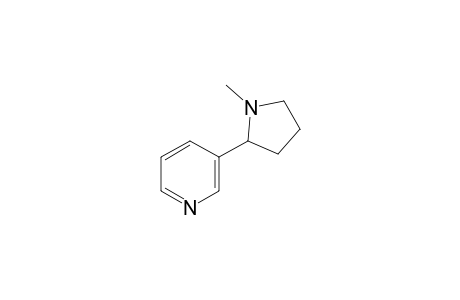  (59)  Nicotine  MW: 162.2 g/mol  [105] | 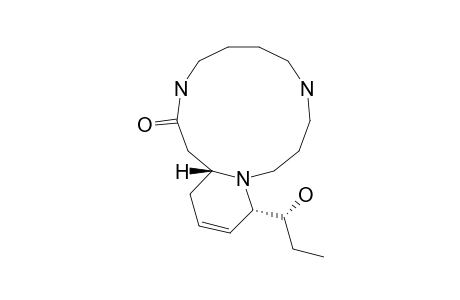  **(60)**  Palustrine  MW: 309.4 g/mol  [105] | 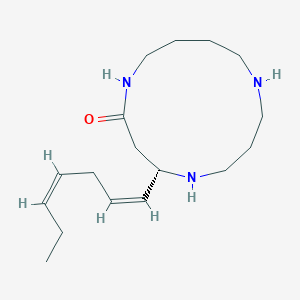  **(61)**  Palustridiene  MW: 293.4 g/mol  [106] |
| 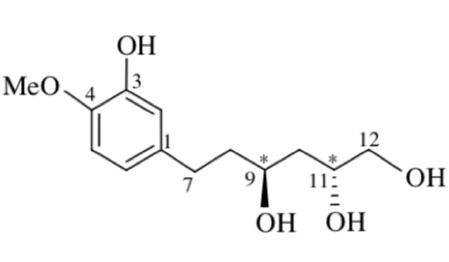  (62)  Phenylhexane debilitriol  MW: 256.2 g/mol  [53] | 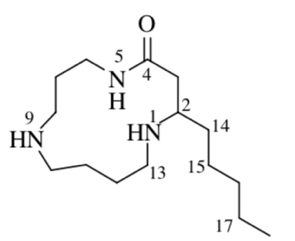  **(63)**  Equisetumine  MW: 269.4 g/mol  [53] | 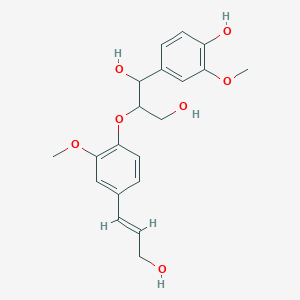  **(64)**  Guaiacylglycerol-β-coniferyl ether  MW: 376.4 g/mol  [106] |
| 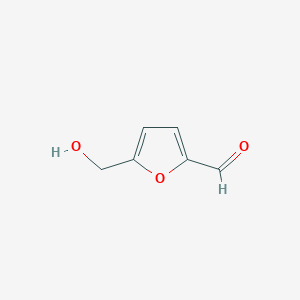  (65)  5-hydroxymethyl-2-furfuraldehyde  MW: 126.1 g/mol  [106] | 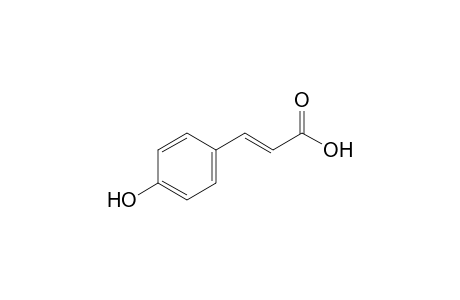  **(66)**  Coumaric acid  MW: 164.1 g/mol  [105] | 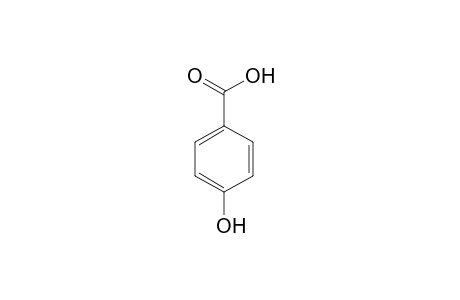  **(67)**  *p*-Hydroxybenzoic acid  MW: 138.1 g/mol  [105] |
| 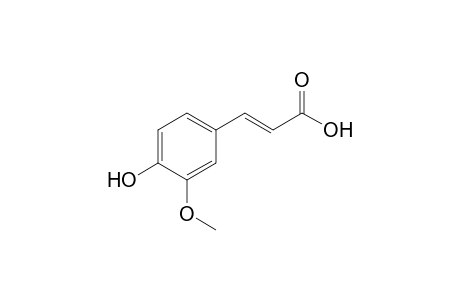  (68)  Ferulic acid  MW: 194.1 g/mol  [105] | 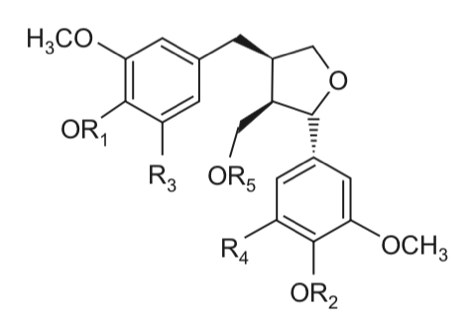  **(69)**  R1-R4= H, R5=Glc  (+)-lariciresinol 9-*O*-β-D-glucopyranoside  MW: 522.5 g/mol  [107] | 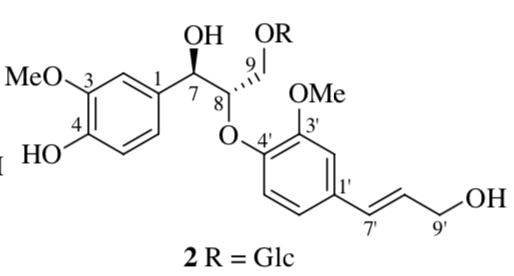  **(70)**  R= Glc  8-*O*-4’ neolignan glucoside debilignanoside  MW: 538.5 g/mol  [53] |
| 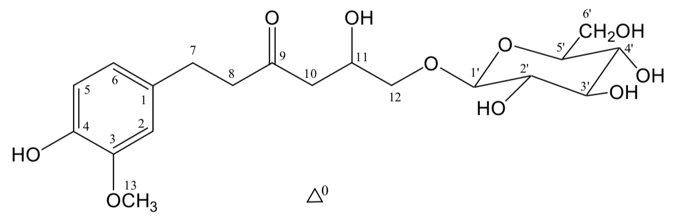  (70)  Equisetumoside B  MW: 416.4 g/mol  [108] | **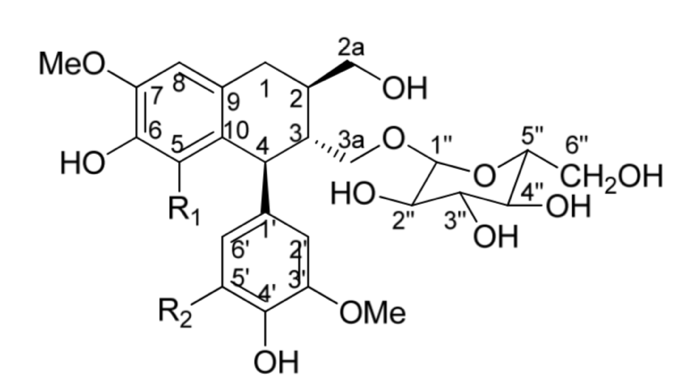(71)**  R1 e R2 = H  (+)-isolariciresinol-3-α-O- β-D-glucopyranoside  MW:522.5 g/mol  [109] | 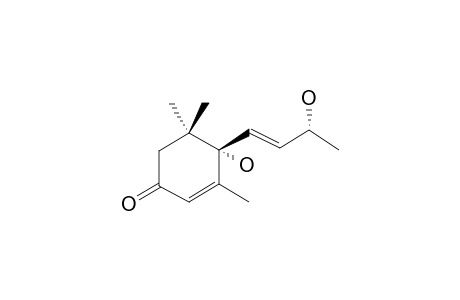  **(72)**  Blumenol A  MW: 224.3 g/mol  [105] |
| 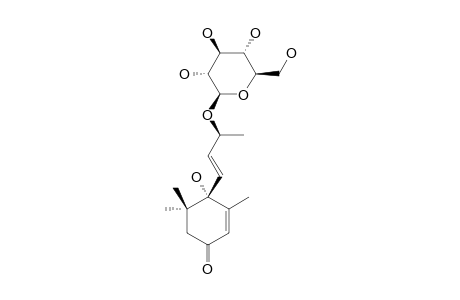  (73)  Corchoinoside C  MW: 386.44 g/mol  [105] | 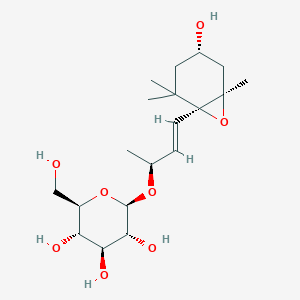  **(74)**  Sammangaoside A  MW: 388.5 g/mol  [106] | 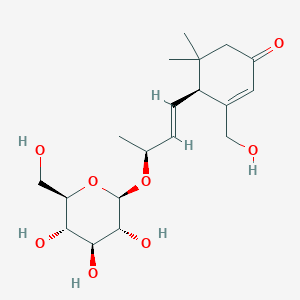  **(75)**  Debiloside A  MW: 386.4 g/mol  [106] |
| 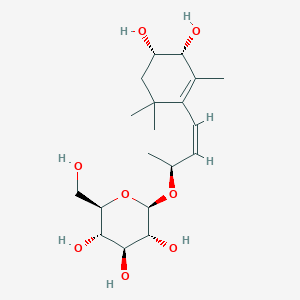  (76)  Debiloside B  MW: 388.5 g/mol  [106] | 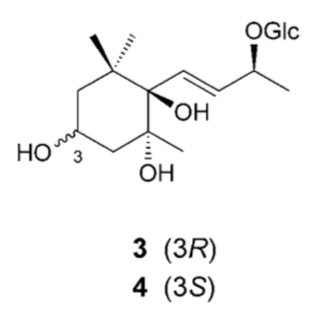  **(77)** Debiloside C (3=R)  **(78)** (3S,5R,6R,7E,9S)‐9‐[(β‐D‐glucopyranosyl)oxy]megastigm‐7‐ene‐3,5,6‐triol (3=S)  MW:406.5 g/mol  [52] | 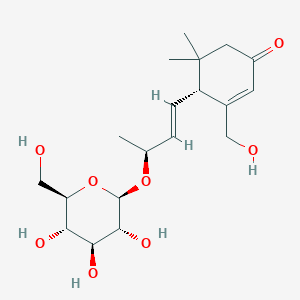  **(79)**  Macarangioside D  MW: 386.4 g/mol  [106] |
| 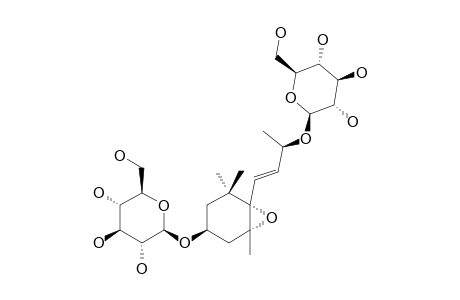  (80)  (3S,5R,6S,7E,9S)-megastigman-7-ene-5,6-epoxy-3,9-diol 3,9-*O*-β-D-diglucopyranoside  MW: 550.6 g/mol  [105] | 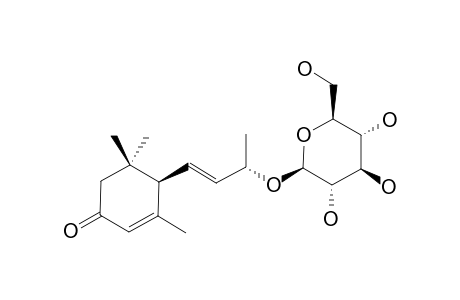  **(81)**  (6R,9S)-3-oxo-α-ionol 9-*O*-β-D-glucopyranoside  MW: 370.4 g/mol  [105] | 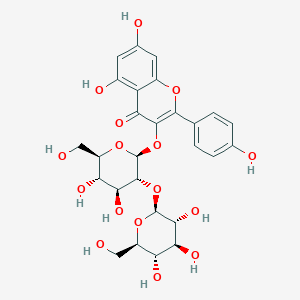  **(82)**  Kaempferol 3-*O*-sophoroside  MW: 610.5 g/mol  [106] |
| 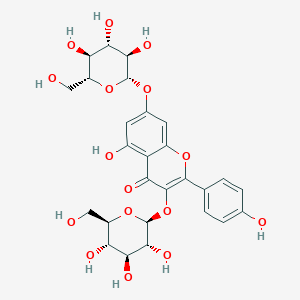  (83)  Kaempferol 3,7-*O*-β-D-diglucopyranoside Syn: Kaempferol 3,7-O-diglucoside  MW: 610.5 g/mol  [106] | 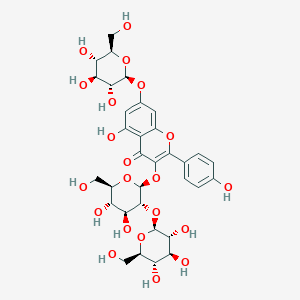  **(84)**  Kaempferol 3-*O*-sophoroside-7-*O*- β-D-glucopyranoside  MW: 772.7 g/mol  [106] | 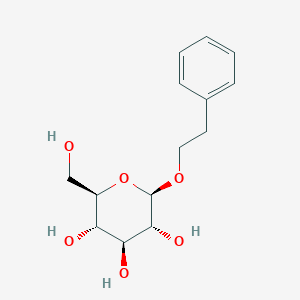  **(85)**  Phenylethyl O- β-D-glucopyranoside  MW: 284.3 g/mol  [106] |
| 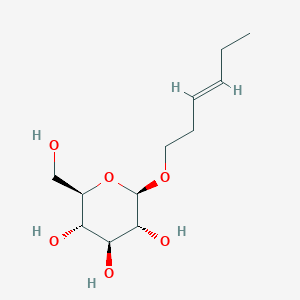  (86)  (Z)-3-hexenyl *O*-β-D-glucopyranoside  MW: 262.3 g/mol  [106] | 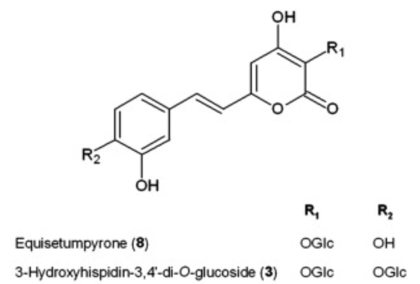  **(87)**  R1= OGlc; R2=OGlc  3-Hydroxyhispidin-3,4′-di-O-glucoside  MW: 587.2 g/mol  [55] | 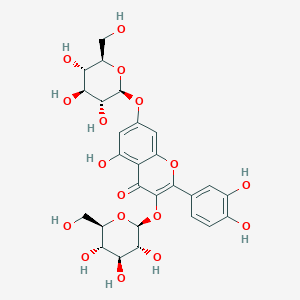  **(88)**  Quercetin 3,7-di-*O-*glucoside  MW: 626.5 g/mol  [106] |
| 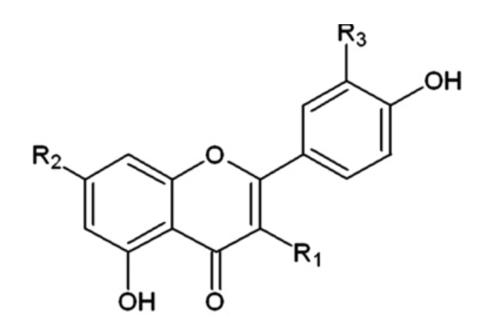  (89)  R1= O-Caff-Glc R2=OH; R3= OH  Quercetin 3-*O*-(caffeoyl)-glucoside  MW: 627.1  [55] | 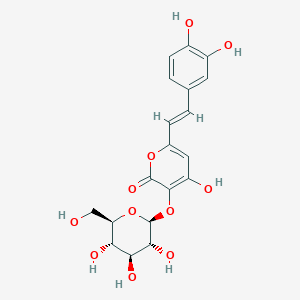  **(90)**  Equisetumpyrone  MW: 424.4 g/mol  [106] | 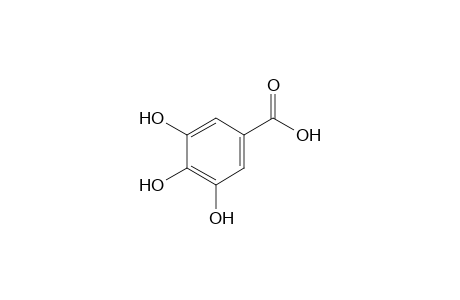  **(91)**  Gallic acid  MW: 170.1 g/mol  [105] |
| 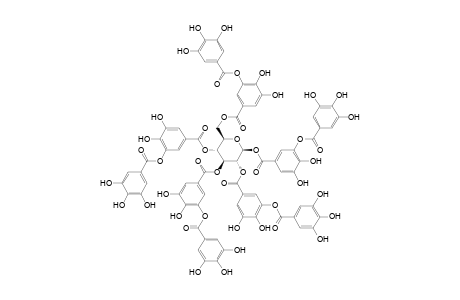  (92)  Tannic acid  MW: 1701.2 g/mol  [105] | 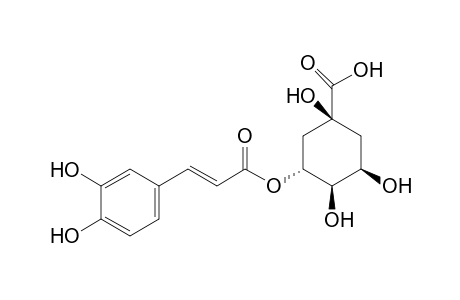  **(93)**  Chlorogenic acid  MW: 354.3 g/mol  [105] | 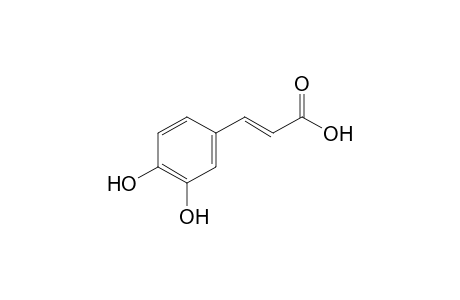  **(94)**  Caffeic acid  MW: 180.1 g/mol  [105] |
| 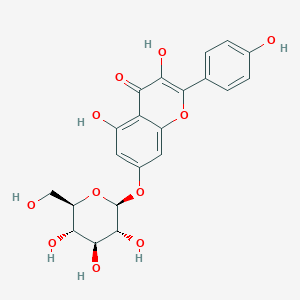  (95)  Kaempferol 7-*O*- β-D-glucopyranoside  MW: 448.4 g/mol  [106] | 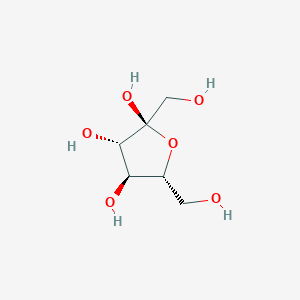  **(96)**  α-D-fructofuranose  MW: 180.1 g/mol  [106] | 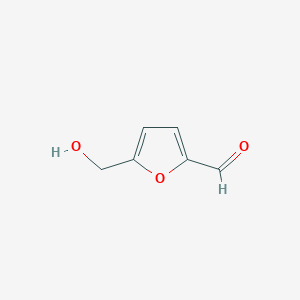  **(97)**  5-hydroxymethylfurfural  MW: 126.1 g/mol  [106] |
| 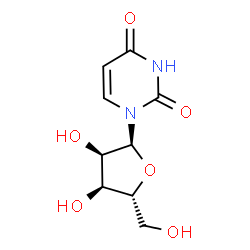  (98)  L-Uridine  MW: 244.0 g/mol  [106] | 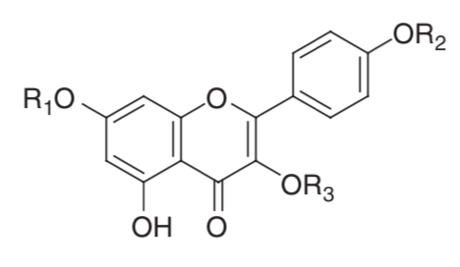  **(99)**  R1= α-L-rhamnoside; R2= β-D-glucopyranoside; R3= H  Kaempferol 7-O-α-L-rhamnoside-4’-O-β-D-glycopyranoside  [57] | 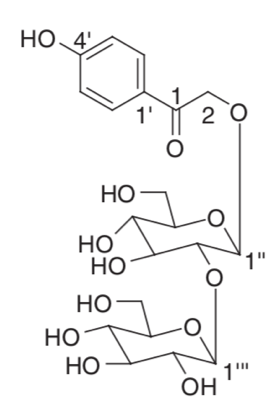  **(100)**  2-(sophorosyl)-1-(4-hydroxyphenyl) ethanone  MW: 476.4 g/mol  [57] |
| 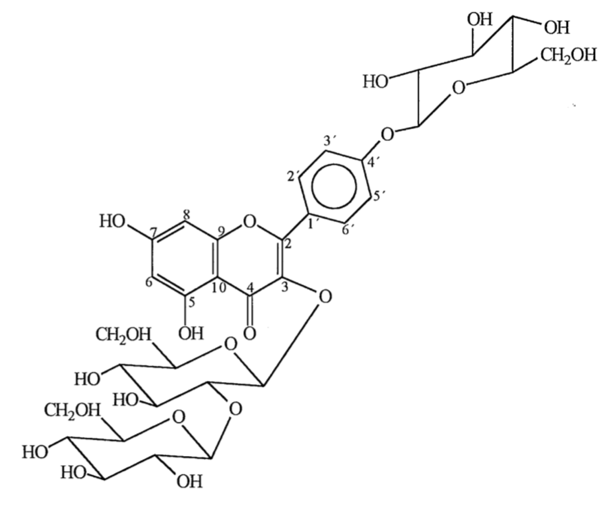  (101)  Kaempferol 3-O-sophoroside-4’-O- β-glucoside  MW: 786.7 g/mol  [58] | **(102)**  Chrysin  MW: 254.2 g/mol  [105] | **(103)**  β-D-glucose  MW: 180.1 g/mol  [106] |
| (104)  N5-formylpalustrine  MW: 337.5 g/mol  [106] | **(105)**  N5-acetylpalustrine  MW: 351.5 g/mol  [106] | **(106)**  18-deoxypalustrine  MW: 293.4 g/mol  [106] |
| (107)  N5 -formylpalustridiene  MW: 321.5 g/mol  [106] | **(108)**  Myricoidine  MW: 293.4 g/mol  [106] | **(109)**  Spermidine  MW: 145.25 g/mol  [106] |
| (110)  Kaempferol 3-O-1”- β-D-glucopyranosyl-3-O-1”’- β -D-glucopyranoside  MW: 610.1 g/mol  [44] | **(111)**  4-*O*-(*p*-coumaroyl)shikimic acid  MW: 320.2 g/mol  [106] | **(112)**  Luteolin-7-O- β-D-glucopyranoside  MW: 448.4 g/mol  [106] |
| (113)  Apigenin-5-*O*- β-D-glucopyranoside  MW: 432.4 g/mol  [106] | **(114)**  R1= H; R2 = OGlu; R3= OH; R4= OGlu  Genkwanin-5-O- β-D-glucopyranoside  [61] | **(115)**  Kaempferol  MW: 286.24 g/mol  [105] |
| (116)  Kaempferol 3’-*O*-rutinoside  MW: 594.5 g/mol  [105] | **(117)**  Kaempferol 3-*O*-rutinoside-7-*O*-sophoroside  MW: 918.8 g/mol  [105] | **(118)**  Phenylacetic acid  MW: 136.1 g/mol  [105] |
| (119)  2-heptenal  MW: 112.1 g/mol  [106] | **(120)**  Heptanoic acid  MW: 130.1 g/mol  [106] | **(121)**  (E)-2-decenal  MW: 154.2 g/mol  [106] |
| (122)  Nonanoic acid  MW:158.2 g/mol  [106] | **(123)**  2,4-decadienal  MW: 152.2 g/mol  [106] | **(124)**  Lauric acid  MW: 200.3 g/mol  [106] |
| (125)  Kaempferol 3-*O*-(6’’-O-acetylglucoside)  MW: 490.4 g/mol  [106]    (128)  Kaempferol 3-O-(6″-O-acetylglucoside)-7-O-rhamnoside  MW: 636.6 g/mol  [106] | **(126)**  Kaempferol 3-*O*-(6’’-*O*-acetylglucoside)-7-*O*-glucoside  MW: 652.6 g/mol  [106]    **(129)**  Kaempferol 3-O-rutinoside-7-O-glucoside  MW: 756.7 g/mol  [106] | **(127)**  Kaempferol 3-*O*-glucoside-7-*O*-rhamnoside  MW: 594.5 g/mol  [106]    **(130)**  Protocatechuic acid  MW: 154.12 g/mol  [106] |
| (131)  Procyanidin dimer B2  MW: 578.5 g/mol  [106] | **(132)**  Procyanidin trimer C1  MW: 866.8 g/mol  [106] | **(133)**  Benzylalcohol  MW: 108.14 g/mol  [106] |
| (134)  β-caryophyllene  MW: 204.3 g/mol  [106] | **(135)**  α-ionone  MW: 192.3 g/mol  [106] | |

**FIGURE S1. Chemical structure of the compounds identified in the *Equisetum* genus. Chemical structures are presented followed by their names, molecular weight and reference.**
